# Factors influencing successful reconstruction of tympanic membrane perforations: a systematic review and meta-analysis

**DOI:** 10.1007/s00405-023-07831-2

**Published:** 2023-02-22

**Authors:** Kata Illés, Dorottya Gergő, Zsuzsanna Keresztély, Fanni Dembrovszky, Péter Fehérvári, András Bánvölgyi, Dezső Csupor, Péter Hegyi, Tamás Horváth

**Affiliations:** 1 Center for Translational Medicine, Semmelweis University, Üllői út 26, Budapest, 1085 Hungary; 2 Department of Otorhinolaryngology, Head and Neck Surgery, Bajcsy-Zsilinszky Hospital, Maglódi út 89-91, Budapest, 1106 Hungary; 3 Department of Pharmacognosy, Semmelweis University, Budapest, Hungary; 4 Institute for Translational Medicine, Medical School, University of Pécs, Pécs, Hungary; 5 Department of Biomathematics and Informatics, University of Veterinary Medicine, Budapest, Hungary; 6 Department of Dermatology, Venereology and Dermatooncology, Faculty of Medicine, Semmelweis University, Budapest, Hungary; 7 Faculty of Pharmacy, Institute of Clinical Pharmacy, University of Szeged, Szeged, Hungary; 8 Institute of Pancreatic Diseases, Semmelweis University, Budapest, Hungary

**Keywords:** Type-I tympanoplasty, Myringoplasty, Prediction, Predictive factors

## Abstract

**Purpose:**

Based on a systematic review and meta-analysis, our study aimed to provide information about the factors that influence the success of tympanic membrane reconstruction.

**Methods:**

Our systematic search was conducted on November 24, 2021, using the CENTRAL, Embase, and MEDLINE databases. Observational studies with a minimum of 12 months of follow-up on type I tympanoplasty or myringoplasty were included, while non-English articles, patients with cholesteatoma or specific inflammatory diseases, and ossiculoplasty cases were excluded. The protocol was registered on PROSPERO (registration number: CRD42021289240) and PRISMA reporting guideline was used. Risk of bias was evaluated with the QUIPS tool. A random effect model was used in the analyses. Primary outcome was the rate of closed tympanic cavities.

**Results:**

After duplicate removal, 9454 articles were found, of which 39 cohort studies were included. Results of four analyses showed significant effects: age (OR: 0.62, CI 0.50; 0.78, *p* value: 0.0002), size of the perforation (OR: 0.52, CI 0.29; 0.94, *p* value: 0.033), opposite ear condition (OR: 0.32, CI 0.12; 0.85, *p* value: 0.028), and the surgeon’s experience (OR: 0.42, CI 0.26; 0.67, *p* value: 0.005), while prior adenoid surgery, smoking, the site of the perforation, and discharge of the ear did not. Four factors: etiology, Eustachian tube function, concomitant allergic rhinitis, and duration of the ear discharge were analyzed qualitatively.

**Conclusions:**

The age of the patient, the size of the perforation, the opposite ear status, and the surgeon’s experience have a significant effect on the success of tympanic membrane reconstruction. Further comprehensive studies are needed to analyze the interactions between the factors.

**Level of evidence:**

Not applicable.

**Supplementary Information:**

The online version contains supplementary material available at 10.1007/s00405-023-07831-2.

## Introduction

The treatment of tympanic membrane perforation is surgical and is done using a graft to reconstruct the tympanic membrane. There are different available surgical techniques, for example, the approach (transcanal, endaural, or retroauricular), the use of a microscope or endoscope, the graft material (fascia, perichondrium, cartilage, fat, etc.), and its position relative to the tympanic membrane (underlay, inlay, or overlay) [1–3]. The success rate is reported to be between 60% and 95% in the literature, and many factors have already emerged that can affect the outcomes [4, 5]. Identifying the real factors that have an impact on success is essential for many reasons: it results in more precise patient education by informing patients of the expected results, it can affect preoperative patient care, and it helps in selecting the proper surgical approach.

Three meta-analyses have been published regarding these predictive factors. However, two of them investigated only the pediatric population [6, 7], while the one published in 2016 investigated both adult and pediatric populations [8]. Since then, several new studies have been performed, investigating even more potential factors with more intention to follow the modern rules of outcome reporting. Our study aimed to provide comprehensive and more accurate information about prediction, including all the new studies and focusing on the patient-related factors, based on a systematic review and meta-analysis. The reason behind focusing on the patient-related factors is that the surgical approach is the choice of the surgeon, while patient-related factors are given. Furthermore, the predictive factors are by definition the characteristics of the patient that affect a particular treatment. Evaluating and comparing a surgical technique requires different scientific questions and approaches. Therefore, we aimed to analyze the predictive factors without evaluating the approaches.

## Methods

Our systematic review was based on the guidance of the Cochrane Handbook [9]. The PRISMA 2020 updated reporting guideline was used for structured manuscript writing [10]. The study protocol was registered in PROSPERO (registration number: CRD42021289240), which we adhered to except for one change: during the pilot period, we decided to exclude experimental studies.

### Eligibility criteria

Type-I tympanoplasty or myringoplasty studies without restriction on sex or age were collected. Exclusion criteria included the following: patients with cholesteatoma, non-type I tympanoplasty procedure, combined procedures, such as cortical mastoidectomy or ossiculoplasty, patients with specific inflammatory diseases (e.g., tuberculosis, and SLE), patients with previous irradiation on the temporal bone and a follow-up period of shorter than 12 months.

With regard to the study type, we accepted only observational studies which investigated the effects of the predictive factors on the outcome of type I tympanoplasty and myringoplasty. Only English language studies were included. We excluded experimental studies, case reports, case series, animal studies, reviews of literature, meta-analyses, and guidelines.

The primary outcome was the success rate, which was defined as closed tympanic cavity at a minimum of 12 months after the surgery. Secondary outcomes were the hearing outcomes, such as the air–bone gap (ABG) and air conduction (AC) difference between the pre- and postoperative hearing status. Hearing was measured in 4 frequencies 0.5 kHz, 1 kHz, 2kHZ and 3 kHz, or, alternatively, the 3 kHz was replaced with the average of 2 kHz and 4 kHz.

### Information sources and search strategy

Our systematic search was conducted on November 24, 2021, and three major databases were used: CENTRAL, EMBASE, and MEDLINE (via PubMed). During the systematic search, the following search key was used without any filters: *myringoplasty OR tympanoplasty*.

The reference lists of the identified studies were reviewed for additional eligible articles.

### Selection process

Three independent review authors (KI, DG and ZSK) performed the selection. After the duplicates were removed, a pilot test was done to refine and clarify the eligibility criteria and to train the reviewers. Management programs (EndNote X9, Clarivate Analytics, Philadelphia, PA, USA) were used for selection. Disagreements after the title and abstract selection were resolved by discussion. The full-text selection was performed by three independent reviewers (KI, DG and ZSK). Disagreements after the full-text selection were resolved by involving a fourth reviewer (TH). Inter-rater reliability with Cohen’s kappa calculation was measured after the title and abstract selection and after the full-text selection.

### Data collection process and data items

From the eligible articles, data were collected by two authors (KI and GD) independently. The following data were extracted from each eligible article: the first author, year of publication, country of origin, study design, basic demographic characteristics (female percentage, age, number of patients), follow-up period, type of surgical procedure, and success rate of the patient groups with or without predictive factors. If it was possible, the data of the secondary outcomes (ABG and AC) were also collected. Disagreements on data extraction were resolved by discussion among the authors.

### Study risk of bias assessment

Two independent authors performed the risk of bias (ROB) assessment independently (KI and ZSK) using the QUIPS risk of the bias assessment tool [11]. Disagreements between the authors were resolved by discussion.

### Synthesis methods

The odds ratio with 95% CI was used for the effect measure; to calculate the odds ratio, the total number of patients in each group and those with the event of interest were extracted from each study. Raw data from the selected studies were pooled using a random effect model with the Mantel–Haenszel method [12–14] and the Hartung–Knapp adjustment [15]. To estimate *τ*^2^ we used the Paule–Mandel method [16], and the Q profile method for calculating the confidence interval of *τ*^2^ [17]. A funnel plot of the logarithm of effect size and comparison with the standard error for each trial was used to evaluate publication bias. Statistical heterogeneity across trials was assessed by means of Cochrane *Q* test, and the *I*^2^ values [18]. *I*^2^ values of 25%, 50%, and 75% were identified as low, moderate and high estimates, respectively. Outlier and influence analyses were carried out following the recommendations of Harrer et al. (2021) and Viechtbauer and Cheung (2010) [17, 19]. Publication bias was assessed with Egger’s test using the Harbord method [20].

## Results

### Search and selection

**Fig. 1:**
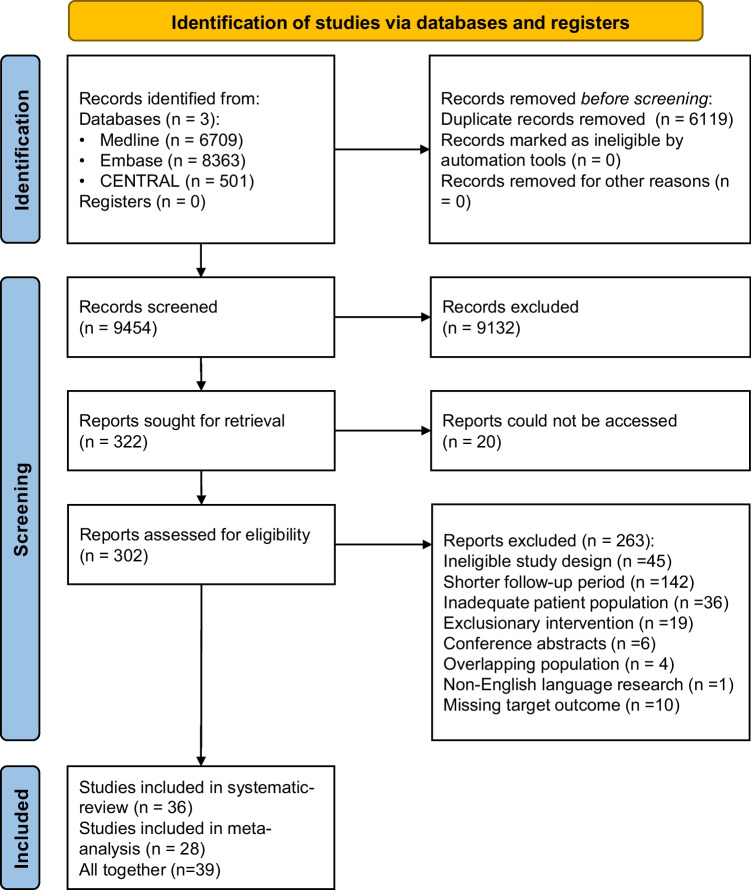
PRISMA 2020 flowchart representing the study selection process

The flowchart of the selection was made according to the PRISMA 2020 reporting guideline [10] (Fig. 1). During the systematic search, 15,573 records were found; this number decreased to 9454 after the duplicate removal. The reviewers identified 322 original studies, of which, at the time of the title and abstract selection, 39 studies were found eligible for inclusion [21–59]. Twenty articles from the original pool could not be found. Two local university libraries were contacted for help, but their search was unsuccessful. The first author (KI) tried to contact the first authors of the missing articles without success.

In total, 28 articles were included in the quantitative and 36 in the qualitative synthesis. The interrater reliability tests were substantial. Cohen’s kappa was 0.72 after the title and abstract selection and 0.71 after full-text selection. No additional studies were found eligible during the reference checking process.

### Basic characteristics of included studies

The characteristics of the eligible studies are detailed in Table 1. From the analyzed studies, the oldest was published in 1970 and the latest in 2021. Three of the studies were prospective and 36 were retrospective. The age distribution of the patients was wide, the range of the mean age was 8.1–48.9 years. The most common surgical technique was the underlay type-I tympanoplasty using temporal fascia graft. However, other types of approach were also used in several articles, see Table 2.

**Table 1:** Characteristics of included studies

Publication data	Study design	Operative technique and material	Demography						
First Author, Publication year			Country	Age (years)		Sex (female% of total)	No of patients	No of operated ear	Follow-up period (months)
				Mean	Range				
Abdelhameed et al. (2017) [21]	Prospective	Underlay, perichondrium	Egypt	19.3	9–65	40	50	50	Min. of 12
Adkins and White (1984) [22]	Retrospective	Underlay, TF	USA	25	4–67	NI	71	71	Min. of 18
Albera et al. (2006) [24]	Inception cohort	Modified overlay or underlay	Italy	37	3–73	58	212	212	Mean: 68
Al-Khtoum and Hiari (2009) [23]	Retrospective	Underlay, TF	Jordan	12	9–14	NI	35	35	Min. of 12
Babu et al. (2019) [25]	RCR	Medial or over-under, TF or perichondrium	USA	24	3–80	NI	95	95	Mean: 25.7
Bajaj et al. (1998) [26]	Prospective	Underlay or overlay	India	NI	5–14	NI	45	45	Min. of 12
Buchwach and Birck (1980) [27]	Retrospective	Underlay, TF	USA	9	3–17	NI	74	80	Mean: 25.2
Callioglu et al. (2016) [28]	Retrospective	Over-underlay, TF or chondro-perichondrial island	Turkey	32.7	NI	53.7	121	121	Mean: 35.76
Caylan et al. (1998) [29]	Retrospective	Underlay, TF	Turkey	NI	5–16	47	51	51	Min. of 18
Dangol and Shrivastav (2016) [30]	Prospective	Underlay, TF	Nepal	26.14	13–62	54.8	219	219	12
Denoyelle et al. (1999) [31]	Retrospective	Underlay, TF	USA	10.5	4–17	43.6	188	188	Mean: 31
Emir (2007) [32]	RCR	Underlay, TF	Turkey	27.4	7-NI	48.6	607	607	Min. of 12
Emmett (1999) [33]	Retrospective	Underlay, perichondrium or TF	USA	NI	< 19	NI	260	260	72–120
Gaslin et al. (2007) [34]	RCR	Cartilage interleave	USA	7.9	3–16	NI	42	42	Mean: 30.3
Gersdorff et al. (1995) [35]	Retrospective	Underlay, TF	Belgium	36	5–73	48	320	320	36
Gianoli (1995) [36]	Retrospective	NI (Wullstein's type I)	USA	8.1	2–16	55	36	36	Mean: 15.3
Gun et al. (2014) [37]	Retrospective	FGM	Turkey	26.4	4–97	47	172	183	12–60
Holmquist (1970) [38]	Retrospective	NI (Wullstein's type I)	Sweden	NI	16–72	NI	120	124	Min. of 24
Iso-Mustajärvi et al. (2018) [39]	Retrospective	Underlay, TF, perichondrium or FGM	Finland	33	4–78	NI	315	315	Mean: 38
Kaya et al. (2018) [40]	Retrospective	Over-underlay, cartilage	Turkey	12.68	9–16	50	76	76	Mean: 76.8
Knutsson et al. (2017) [41]	Retrospective	FGM	Norway	30.4	4–85	43	100	100	12
Lee et al. (2016) [42]	Retrospective	Paper patch myringoplasty	Republic of Korea	41.6	NI	52.6	114	114	Min. of 12
Li et al. (2020) [43]	Retrospective	Underlay, TF	China	45.92	19–68	56.6	53	53	12–24
Lou (2021) [44]	Retrospective	Underlay, cartilage	China	48.87	NI	40	131	131	24
Migirov et al. (2013) [45]	Retrospective	Underlay, TF or tragal perichondrium	Israel	35.9	18–79	63	65	65	Min. of 12
Ophir et al. (1987) [46]	Retrospective	Underlay, overlay, TF	Israel	NI	5–12	43.9	155	172	Mean: 38.4
Övet et al. (2016) [47]	Retrospective	Overlay–Underlay, chondro-perichondrial island	Turkey	Children: 9 adults: 30	Children: 8–16 adult: 19–64	53	133	133	24
Podoshin et al. (1996) [48]	Retrospective	NI (Wullstein's type I, TF)	Israel	12	9–14	NI	51	51	24–108
Salvador et al. (2021) [49]	RCR	Underlay, TF	Portugal	39.7	18–69	57	155	155	Mean: 15.6
Sengupta and Kacker (1974) [50]	Retrospective	NI	India	NI	NI	NI	104	104	12–24
Shankar et al. (2015) [51]	Prospective	Over-underlay, TF	India	NI	15–45 +	44	70	70	Mean: 14.4
Strahan et al. (1971) [52]	Retrospective	Medial or lateral technique, TF, perichondrium, or vein	USA	NI	> 10–70	38	483	483	Mean: 18.8
Takahashi-Tatsumi et al. (2014) [53]	Retrospective	NI	Japan	Children: 8.7 adult: 47.1	Children: 2–16 adult: NI	46	130	130	Mean: 40.7
Tseng et al. (2018) [54]	Retrospective	Underlay, TF, or perichondrium	Taiwan	51.7	18–86	51	181	181	Mean: 15.4
Ullah et al. (2008) [55]	Retrospective	Underlay or overlay,TF	Pakistan	10	8–14	44	100	100	36
Vartiainen and Nuutinen (1993) [56]	Retrospective	Underlay or lateral graft, TF	Finland	NI	> 10–60 +	NI	404	404	Mean: 66
Vartiainen and Vartiainen (1997) [57]	Retrospective	Underlay or lateral graft, TF	Finland	11.1	5–17	55	60	60	Min. of 60
Westerberg et al. (2011) [58]	RCR	Underlay, TF	Sweden	29	3–82	45.7	232	243	12–60
Yung et al. (2007) [59]	Retrospective observational	Underlay, TF, perichondrium, or cartilage	UK	NI	4–13	NI	54	54	12–36

*N*^*0*^ number, *NI* no information, *Min.* minimum, *USA* United States of America, *UK* United Kingdom, *RCR* retrospective chart review, *TF* temporalis fascia, *FGM* fat graft myringoplasty

**Table 2:** Pooled outcomes of ten predictive factor analyses

Outcomes		No of the included studies	Total No of included patients	Group of patients with the predictive factor		Comparator group		Overall OR	CI	*p* value	Heterogeneity	
Factors	Comparators			No of the success	Total	No of success	Total				*I*^2^	CI
**Age: under 16** **years**	**Age: over 16** **years**	7	1798	335	401	1229	1397	**0.62**	**0.50; 0.78**	**0.0002**	0%	0%; 71%
Age: under 8 years	Age: over 8 years	4	309	85	111	155	198	0.89	0.75; 1.06	0.119	0%	< 0%; < 85%
No prior surgery	Prior adenectomy or adenotonsillectomy	4	411	247	286	108	125	1.14	0.42; 3.11	0.698	0%	0%; 85%
**Size of the perforation over 50%**	**Size of the perforation under 50%**	13	2207	657	783	1270	1424	**0.52**	**0.29; 0.94**	**0.033**	51%	8%; 74%
Site: anterior	Site: posterior	7	405	234	270	126	135	0.52	0.11; 2.52	0.338	52%	0%; 81%
Site: marginal	Site: central	3	643	87	98	467	545	0.99	0.14; 7.09	0.984	9%	0%; 90%
Operated ear status: wet/discharging	Operated ear status: dry	9	1952	267	307	1400	1645	0.95	0.57; 1.57	0.39	5%	0%; 67%
**Opposite ear: diseased**	**Opposite ear: normal**	9	1005	267	384	514	621	**0.32**	**0.12; 0.85**	**0.028**	73%	46%; 86%
Patients: smoker	Patients: non-smoker	3	439	51	68	310	371	0.50	0.11; 2.39	0.198	8%	0%; 90%
**Surgeon experience: resident**	**Surgeon experience: senior**	6	1559	733	869	639	690	**0.42**	**0.26; 0.67**	**0.005**	0%	0%,75%

*CI* confidence interval, *N*^*0*^ number, OR odds ratio

The statistically significant outcomes (*p* value <0.05) are bolded

### Quantitative synthesis

To make more homogenous groups, the fat graft and paper patch myringoplasties were not included in the quantitative synthesis.

**Fig. 2:**
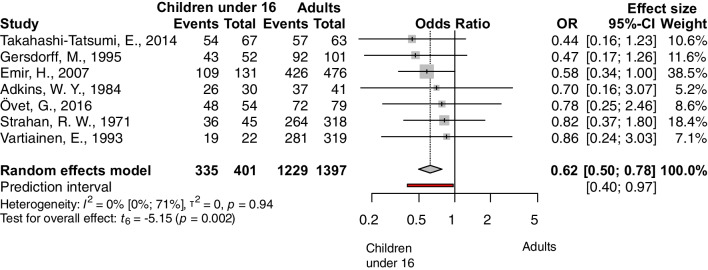
Forest plot presenting the pooled odds of success after tympanic membrane reconstruction: comparison between the pediatric population under 16 years of age and adults

**Fig. 3:**
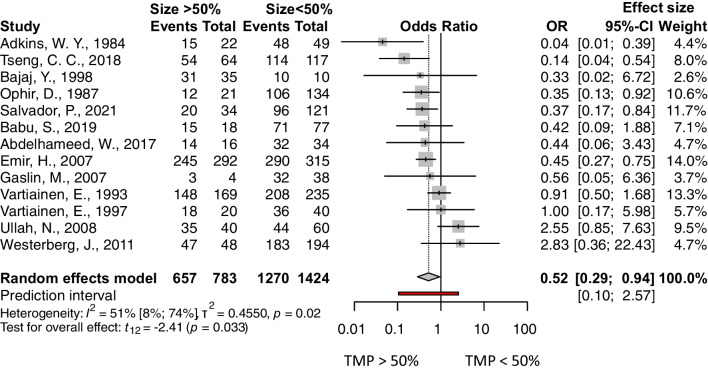
Forest plot presenting the pooled odds of success after tympanic membrane reconstruction: comparison between perforation sizes over 50% to under 50%

**Fig. 4:**
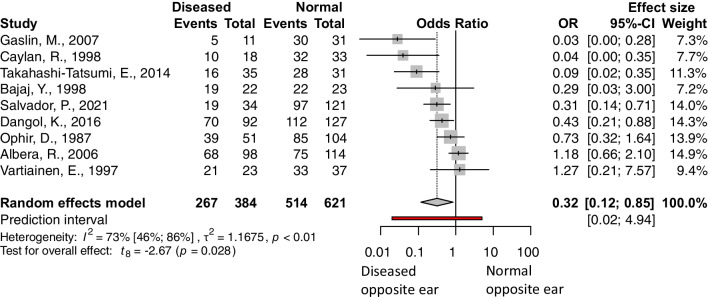
Forest plot presenting the pooled odds of success after tympanic membrane reconstruction: comparison between patients with normal opposite ear status and patients with diseased opposite ear status

**Fig. 5:**
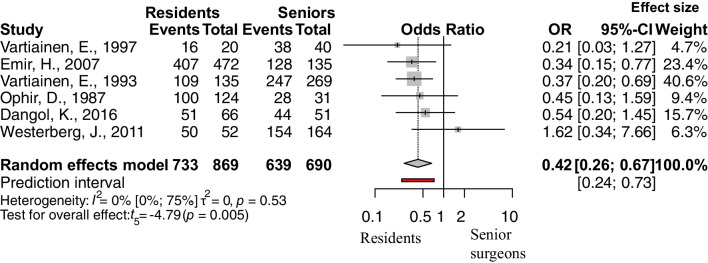
Forest plot presenting the pooled odds of success after tympanic membrane reconstruction: comparison between experience of the surgeons

The following factors were analyzed: age, separating adult and pediatric population (under vs. over 16 years), age among children (under vs. over 8 years), presence of prior adenectomy or adenotonsillectomy vs. no prior surgery, size of the perforation (perforation affecting more than 50% of the tympanic membrane vs. less than 50%), site of the perforation 1 (central or marginal), site of the perforation 2 (anterior or posterior), condition of the operated ear (discharging/wet ears vs. dry), condition of the opposite ear (diseased or normal), experience of the surgeon (senior or resident), and smoking status. The outcome measurement was the success rate, and the odds of success were calculated. The pooled results can be seen in Table 2 and the detailed Forest plots are shown in Figs. 2, 3, 4 and 5 and Supplementary Figs. 6–11/A.

Four factors were found statistically significant: patients under vs. over 16 years of age (OR: 0.62, CI 0.50; 0.78, *p* value: 0.0002), see Fig. 2, the size of the perforation (OR: 0.52, CI 0.29; 0.94, *p* value: 0.033), see Fig. 3, the condition of the opposite ear (OR: 0.32, CI 0.12; 0.85, *p* value: 0.028), see Fig. 4, and the experience of the surgeon (OR: 0.42, CI 0.26; 0.67, *p* value: 0.005), see Fig. 5.

In addition, when the age group of children was targeted, no significant result was found (OR: 0.89, CI: 0.75; 1.06 *p* value: 0.119), see Suppl. Fig. 3/A. The odds for success were 11% lower in children under 8 years compared to those over 8 years.

We could not detect an effect regarding the condition of the ear (wet vs. dry) at the time of the surgery (OR: 0.95, CI 0.57; 1.57, *p* value: 0.39); nor was an effect detected regarding prior adenectomy or adenotonsillectomy vs. no prior surgery outcome (OR: 1.14, CI 0.42; 3.11, *p* value: 0.6989), see Suppl. Figs. 4/A and 5/A.

Regarding the site of the perforation, neither the comparison of the anterior and posterior perforations (OR: 0.52, CI 0.11; 2.52, *p* value: 0.338) nor the comparison of the central and marginal perforations were found statically significant (OR: 0.99, CI 0.14; 7.09 *p* value: 0.984), see Suppl Figs. 5/A and 6/A.

The comparisons between the smoker and non-smoker groups found an effect (OR: 0.50, CI 0.11; 2.39, *p* value: 0.198), although it was not statically significant, see Suppl Fig. 8/A.

### Risk of bias assessment

The results of the risk of bias assessments are presented in Supplementary Fig. 1. The overall risk of bias in the included studies for success rate outcome was 50% low, 36.8% medium and 13.2% high. Articles with high ROB were not included in the quantitative analyses. Regarding the hearing outcome, the overall risk of bias was 81% low, 9.5% medium and 9.5% high.

### Publication bias

Publication bias was visualized with contour-enhanced funnel plots (Supplementary Figs. 2 and 3/B–8/B). For one outcome, (the size of the perforation) where more than ten articles were included in the analysis, Egger’s test was calculated. It gave a *p* value of 0.798 (*t*: − 0.34, *df*: 11) indicating no publication bias.

### Qualitative synthesis

We were not able to create a mathematical analysis for four factors: the etiology of the perforation [39, 56, 58], Eustachian tube function [38, 43, 48], concomitant allergic rhinitis [28], and duration of the ear discharge [23, 26].

The studies that evaluated the etiology of the perforation used different classifications, and did not find any clear relationship between the etiology and the outcomes [56].

There are no routinely accessible objective and reliable measuring methods to evaluate Eustachian tube function. However, the dysfunction can be estimated with different approaches. In three studies, three different methods were used [38, 43, 48]. Their data showed a connection between the decreased success of type I tympanoplasty and Eustachian tube dysfunction.

One study investigated the connection between allergic rhinitis and surgical success, but it could not confirm a significant difference[28].

Two studies investigated the duration of the ear discharge before the surgery. Their data suggested that a long duration of discharge influences the graft uptake negatively[23, 26].

### Fat graft and paper patch myringoplasty

Two articles investigated the predictive factors of fat graft myringoplasty (FGM) [37, 41] and one article investigated paper patch myringoplasty [42]. These methods differ from traditional techniques, because the tympanomeatal flap is not elevated during the procedure. Therefore, we decided to make a separate section for these interventions.

The two studies which investigated FGM did not find a statistically relevant difference in terms of location, size, and age at 1 year of follow-up [37, 41]. However, the size of the perforation of the targeted population was limited to small and medium sizes. Three predictors were found to be decisive in paper patch myringoplasty in one study: the patient’s age, etiology of the perforation, and history of otorrhea [42].

### Hearing outcomes

In most of the cases, hearing improved after surgery. According to data from 14 articles, the average ABG improvement was 10.46 dB (range 5.6–18.83 dB) [21, 24, 25, 29–31, 34, 39, 40, 43, 44, 47, 49, 54, 58]. The improvement of AC was 11.26 dB (range 8.4–17 dB) [24, 29–31, 39, 47, 49, 58] in eight articles, see Table 3. There is a correlation between anatomic success and hearing improvement: if the success of the surgery is high, hearing improvement will follow the outcome, but it will not reach the same level of improvement.

**Table 3:** Pooled Air Bone Gap (ABG) and Air Conduction (AC) difference before and a minimum of 12 months after the surgery

First Author, Publication year	ABG difference (dB)	AC difference (dB)
Abdelhameed et al. (2017) [21]	9.9	NR
Albera et al. (2006) [24]	8	10
Babu et al. (2019) [25]	11.8	NR
Caylan et al. (1998) [29]	13.5	15
Dangol and Shrivastav (2016) [30]	9.8	11.44
Denoyelle et al. (1999) [31]	10.85	10.85
Iso-Mustajärvi (2018) [39]	5.6	NR
Kaya et al. (2018) [40]	16.22	17
Li et al. (2020) [43]	6.8	NR
Lou (2021) [44]	18.83	NR
Övet et al. (2016) [47]	7.37	9.97
Salvador et al. (2021) [49]	7.42	7.44
Tseng et al. (2018) [54]	11.5	NR
Westerberg et al. (2011) [58]	8.6	8.4
MEAN	10.46	11.2625

*ABG* air bone gap, *AC* air conduction, *dB* decibel

## Discussion

Ten factors that appeared in at least three different studies with similar methods were identified and analyzed. Out of the ten results, four were statistically significant and the effects were robust (Table 2). These factors were age, size of the perforation, condition of the opposite ear, and the experience of the surgeon. In these results, heterogeneity was low or moderate (Suppl. Figs. 2–11). This suggests that these four factors influence the success of tympanoplasty. However, the interaction between these factors is unrevealed; other factors may also have a significant effect, although the sample size may be too low, or other factors may lessen the effects.

The previous metanalyses investigated similar factors (age, size of the perforation, condition of the contralateral and the operated ear, previous adenectomy etc.) [6–8], but none of them investigated the influence of the surgeon experience and the smoking status of the patients. Furthermore, our investigation used a rigorous inclusion criteria, and only studies with minimum of 12 month follow-up were included. Fifteen of the included studies was published since the last meta-analysis’s systematic search, July, 2014 [21, 25, 28, 30, 37, 39, 40, 42–44, 47, 49, 51, 53, 54]. Besides, one of the pediatric meta-analyses found only the age as a significant factor [7], the other one found only the size of the perforation and the condition of the contralateral ear significant [6]. The most similar study to ours, where adult and children were included, found the larger perforations and pediatric population as a negative influencer [8].

### Age

We have found a total of 21 articles in which the age of the patient was evaluated as a predictive factor [22–27, 29, 32, 33, 35, 40, 46–48, 52–57, 59]. However, there is no consensus on how to define the age groups: some studies consider individuals over 16 years of age as adults, and some others over 18. We set the age limit for inclusion to 16 years, as most articles do. By applying this limit, the comparison of adults and children gave clear and homogenous results from seven article [22, 32, 35, 47, 52, 53, 56], suggesting that the children have lower odds of success at 50% (Fig. 2). We further analyzed the data in the pediatric population of four elidable articles [26, 27, 46, 59], and we compared children under and above 8 years of age. The result was not significant; however, some effect was detected (Suppl. Fig. 3/A). One of the previous meta-analyses could not confirm a significant difference in age-specific or indexed age analyses [6], while the other found a difference in success when younger children were compared with older ones [7]. It must be mentioned that our pediatric population was small, and the confidence intervals were wide. It is assumed that other factors also had a distorting effect.

### Size of the perforation

Regarding the size of the perforation, we found a significant and strong correlation after analysing 13 studies' data [21, 22, 25, 26, 32, 34, 46, 49, 54–58]. If the size of the perforation is larger than 50% of the tympanic membrane, it is thought to decrease the odds of success (Fig. 3). The heterogeneity of the result is moderate, 51%, which could originate from the hard separation.

### Condition of the opposite ear

Nine studies reported data about the condition of the opposite ear, which this could mean perforation, effusion, cholesteatoma, etc. [24, 26, 29, 30, 34, 46, 49, 53, 57]. The reason behind the pooling of the ear problem is the assumed Eustachian tube dysfunction. Although the heterogeneity of the result is high due to the pooling of different ear problems, the result is significant, and the effect is the most marked of all our results (Fig. 4).

### Experience of the surgeon

Although the experience of the surgeon is not a real patient-related factor, it is a given situation from the perspective of the patient. Six articles reported information about the experience of the surgeon [30, 32, 46, 56–58] (Fig. 5). The result with low heterogeneity suggests that the patients of resident doctors have significantly lower odds of success than the patients of senior surgeons. This strong correlation suggest that the personal factors should not be forgotten.

### Condition of the operated ear

Nine studies have reported data about the operated ear status [22, 24, 29, 30, 32, 46, 49, 51, 56]. The target of the comparison was the dry ear at the time of the operation vs. discharging/wet ear at the time of the operation. The odds and the confidence interval of the analyses were around 1; therefore, no relevant effect was detected (Suppl. Fig. 4/A).

### Site of the perforation

We performed two analyses regarding the site of the perforation, comparing marginal perforations with central ones [49, 56, 58] (Suppl. Fig. 5/A), and anterior perforations with posterior ones [22, 25, 30, 35, 49, 54, 58] (Suppl. Fig. 6/A). None of the results was significant with low number of included patients. When marginal perforations were compared with central perforations, no effect was found, but the anterior perforation decreased the overall odds ratio to 0.52 compared to posterior perforations. Although  the result was not significant, the anterior perforations are more difficult to assess and operate; therefore, there is a clinically relevant difference between the anteriorly and posteriorly located perforations.

### Prior adenectomy or adenotonsillectomy

Four studies included in their reported data the information about prior adenectomy or adenotonsillectomy [44, 46, 48, 57]. The reason behind the comparison is that frequent inflammation around the Eustachian tube may affect the success of tympanoplasty, which was not proved by our results (Suppl. Fig. 7/A).

### Smoking status

Three studies reported data about the smoking status of the patient [30, 45, 49] (Suppl. Fig. 8/A). Although the result was not considered significant with low number of included patients, there was a detectable effect. One of the three studies had an overall OR over one with a wide confidence interval[45], but the other two suggest that the habit of smoking negatively influences the odds of success[30, 49]. In addition, previous review also pointed out, in the case of smokers the success rate may be less than in non-smokers [60].

### Strengths and limitations

The strengths of our study are the rigorous and state-of-the-art methodology, the high number of included studies and the high number of analyzed factors. We included studies with a minimum follow-up of 12 months with a low or medium risk of bias.

With regard to the limitations of our study, there is heterogeneity in the applied surgical techniques. The lack of data did not allow us to perform a multivariate analysis; therefore, the connections between the factors remain unrevealed.

### Implications for practice and research

Further studies are needed for both the significant and questionable factors with large sample sizes, and they should be analyzed with multilevel methods.

The early application of research results in practice is essential for more efficient health care [61, 62]. For middle ear surgeons, our results are important regarding patient education and for decision-making about the intervention. High-risk patients (children with large perforations and diseased opposite ears) need experienced surgeons who can tailor the appropriate technique to repair the perforation.

## Conclusion

According to our results, four factors have a significant effect on the success rate of tympanic membrane reconstruction: age of the patients, size of the perforation, opposite ear status, and the experience of the surgeon. However, discharging operated ear, site of the perforation, prior adenectomy or adenotonsillectomy and smoking status of the patients were not found significant. Further comprehensive studies are needed to analyze the interactions between the factors to create reliable predictions.

## Supplementary Information

Below is the link to the electronic supplementary material.

Supplementary file1 (PPTX 799 KB)

## Data Availability

The data that support the findings of this study are available from the corresponding authors [TH and KI], upon reasonable request.
