# Supplementary material for: Factors influencing successful reconstruction of tympanic membrane perforations: a systematic review and meta-analysis
Source: Eur Arch Otorhinolaryngol. 2023 Feb 22;280(6):2639–52. doi: 10.1007/s00405-023-07831-2 (PMC10175362; doi:10.1007/s00405-023-07831-2)
Supplement: Supplementary file 1 — Supplementary file1 (PPTX 799 KB) [file 405_2023_7831_MOESM1_ESM.pptx]

## Slide 1
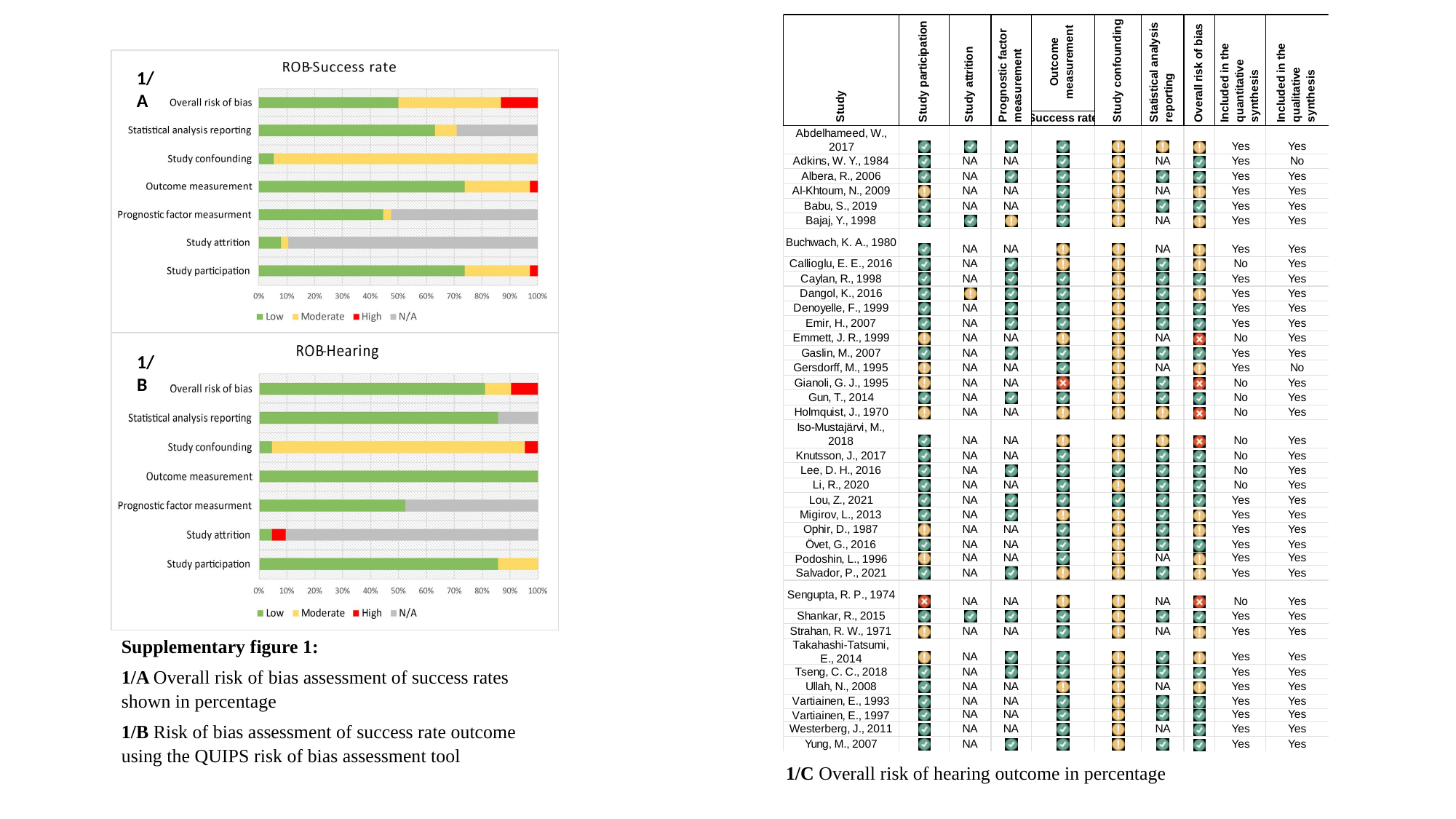

1/A
1/B
Supplementary figure 1:
1/A Overall risk of bias assessment of success rates shown in percentage
1/B Risk of bias assessment of success rate outcome using the QUIPS risk of bias assessment tool
1/C Overall risk of hearing outcome in percentage

## Slide 2
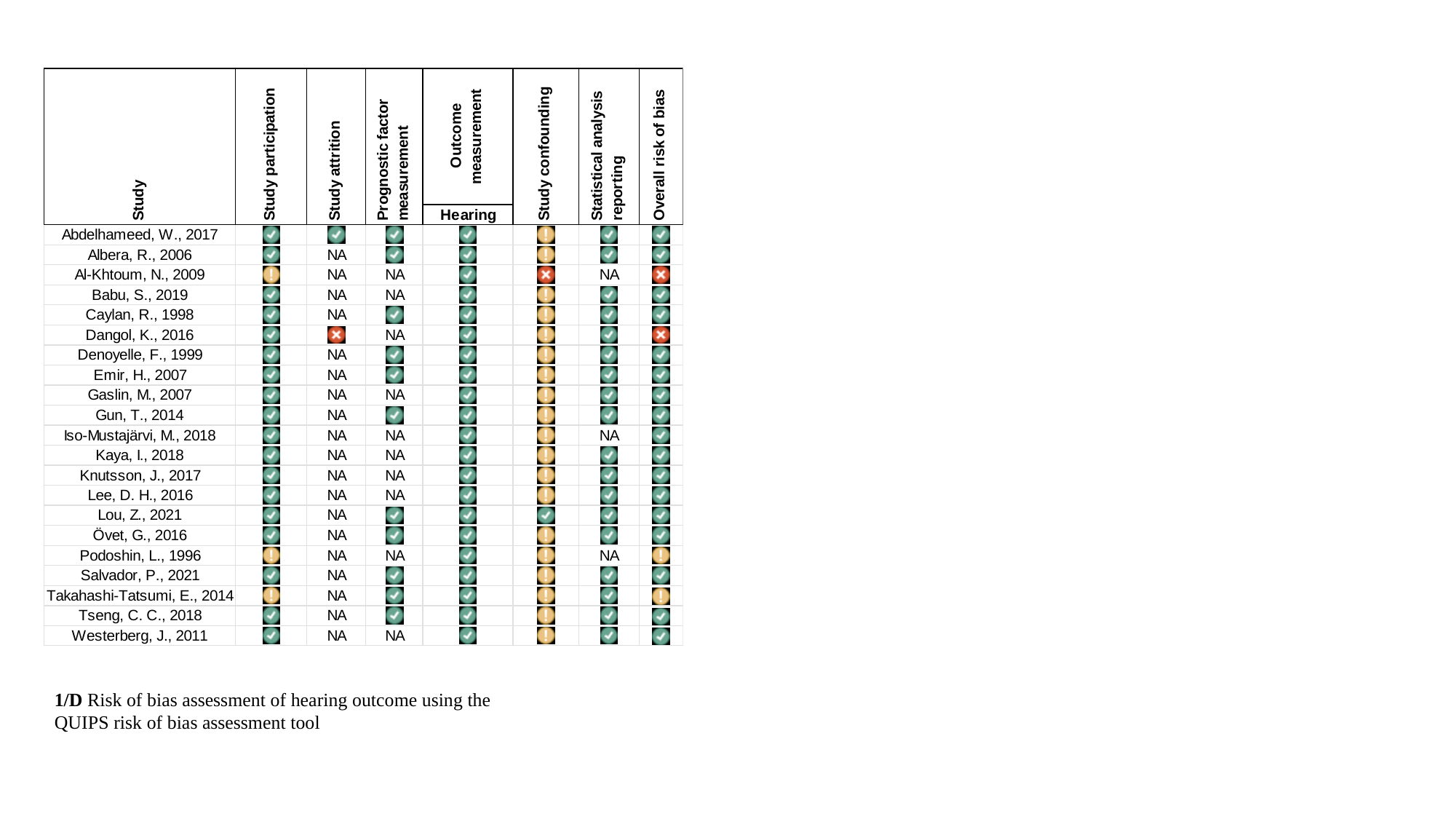

1/D Risk of bias assessment of hearing outcome using the QUIPS risk of bias assessment tool

## Slide 3
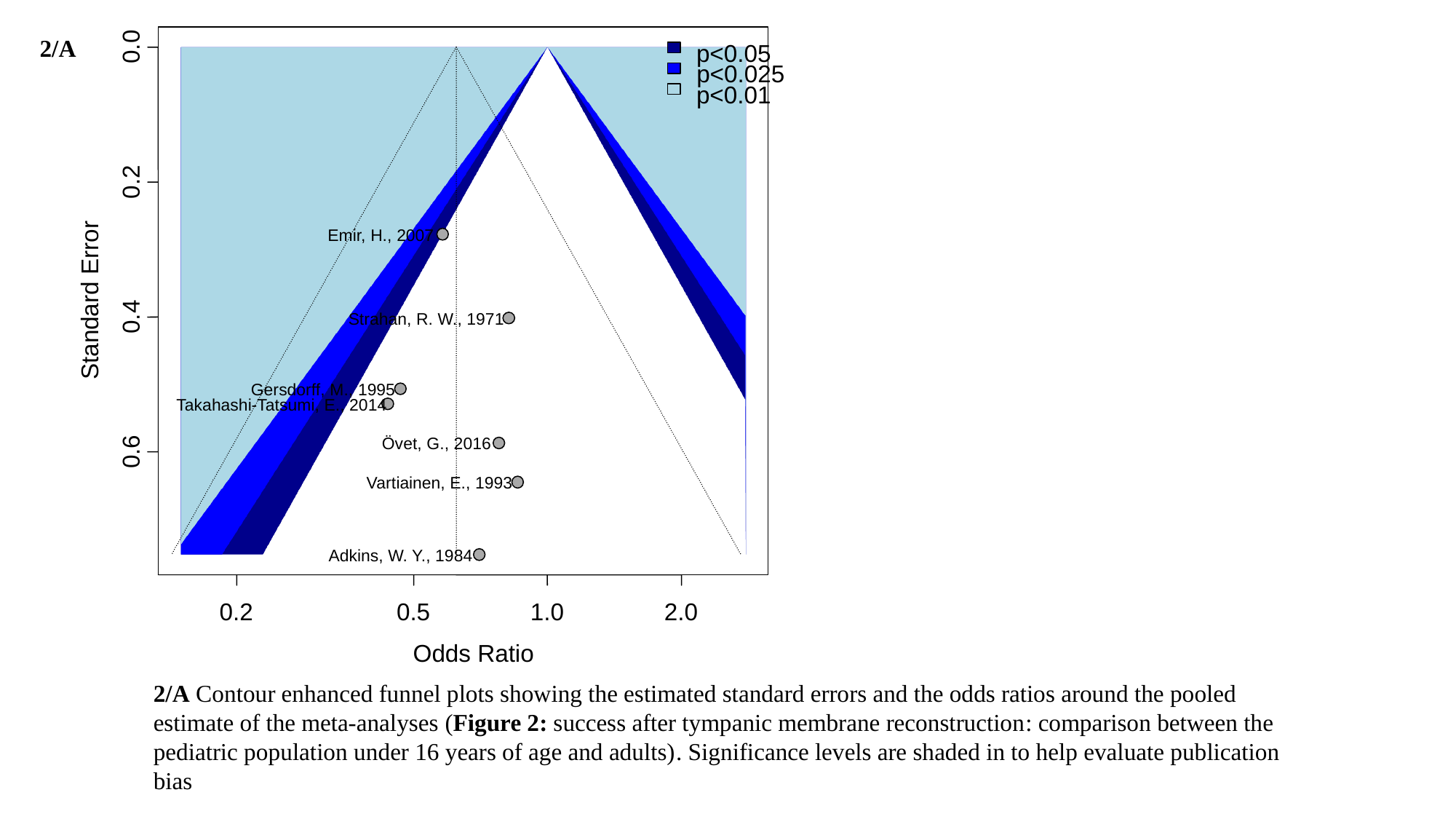

0.0
p<0.05
p<0.025
p<0.01
0.2
Emir, H., 2007
Standard Error
0.4
Strahan, R. W., 1971
Gersdorff, M., 1995
Takahashi-Tatsumi, E., 2014
Övet, G., 2016
0.6
Vartiainen, E., 1993
Adkins, W. Y., 1984
0.2
0.5
1.0
2.0
Odds Ratio
2/A
2/A Contour enhanced funnel plots showing the estimated standard errors and the odds ratios around the pooled estimate of the meta-analyses (Figure 2: success after tympanic membrane reconstruction: comparison between the pediatric population under 16 years of age and adults). Significance levels are shaded in to help evaluate publication bias

## Slide 4
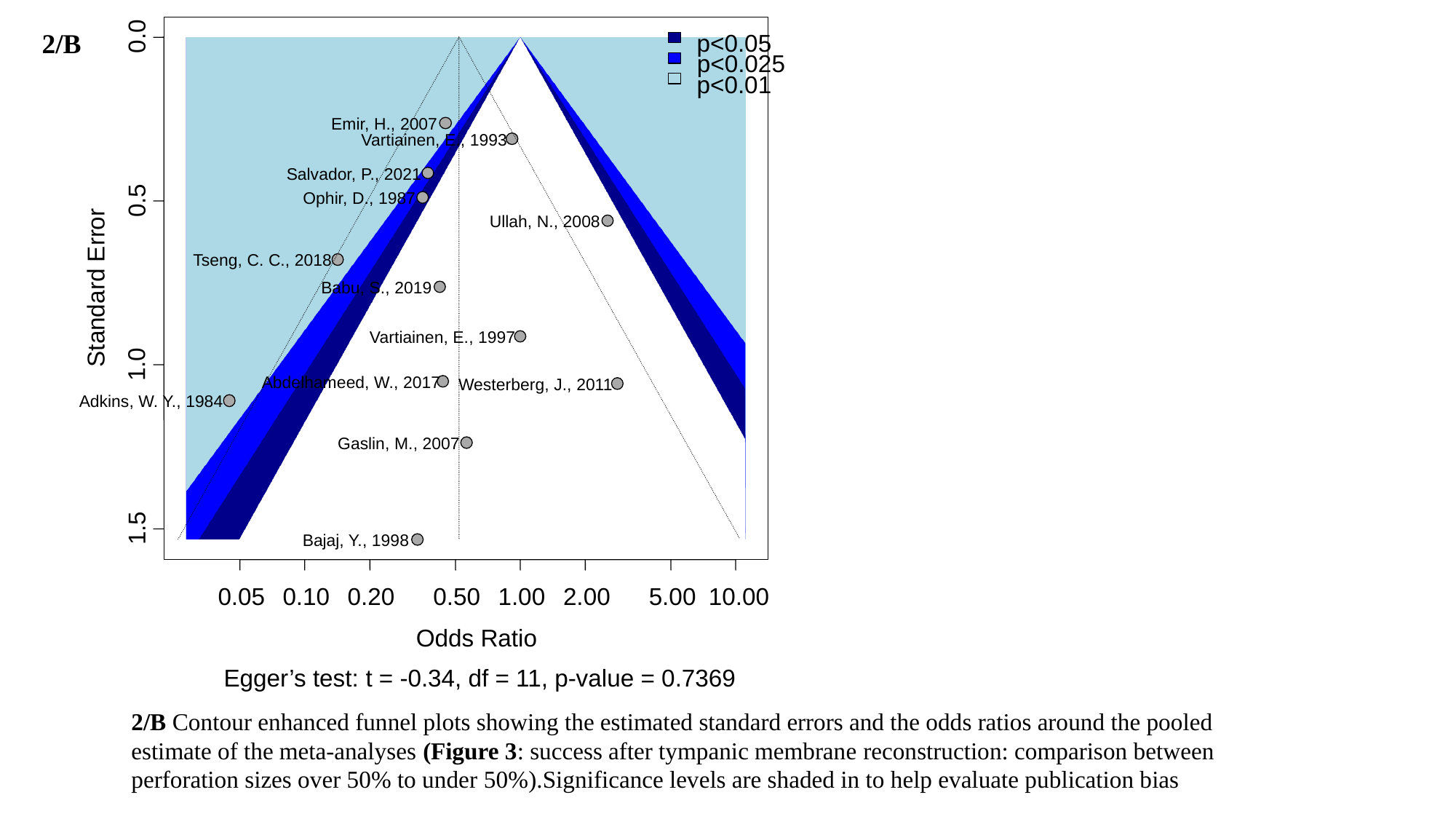

0.0
p<0.05
p<0.025
p<0.01
Emir, H., 2007
Vartiainen, E., 1993
Salvador, P., 2021
0.5
Ophir, D., 1987
Ullah, N., 2008
Tseng, C. C., 2018
Standard Error
Babu, S., 2019
Vartiainen, E., 1997
1.0
Abdelhameed, W., 2017
Westerberg, J., 2011
Adkins, W. Y., 1984
Gaslin, M., 2007
1.5
Bajaj, Y., 1998
0.05
0.10
0.20
0.50
1.00
2.00
5.00
10.00
Odds Ratio
2/B
Egger’s test: t = -0.34, df = 11, p-value = 0.7369
2/B Contour enhanced funnel plots showing the estimated standard errors and the odds ratios around the pooled estimate of the meta-analyses (Figure 3: success after tympanic membrane reconstruction: comparison between perforation sizes over 50% to under 50%).Significance levels are shaded in to help evaluate publication bias

## Slide 5
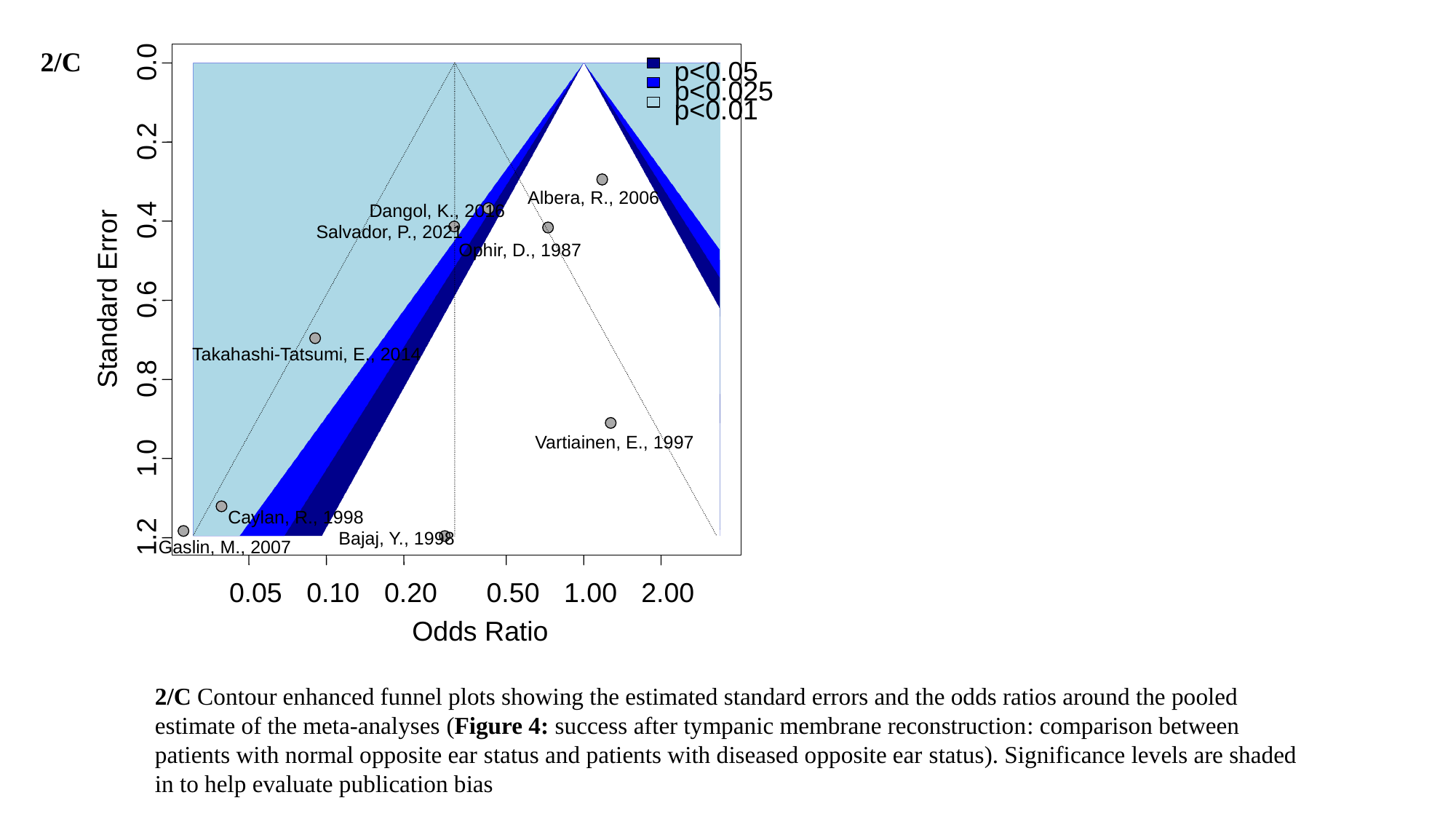

0.0
p<0.05
p<0.025
p<0.01
0.2
Albera, R., 2006
Dangol, K., 2016
0.4
Salvador, P., 2021
Ophir, D., 1987
Standard Error
0.6
Takahashi-Tatsumi, E., 2014
0.8
Vartiainen, E., 1997
1.0
Caylan, R., 1998
1.2
Bajaj, Y., 1998
Gaslin, M., 2007
0.05
0.10
0.20
0.50
1.00
2.00
Odds Ratio
2/C
2/C Contour enhanced funnel plots showing the estimated standard errors and the odds ratios around the pooled estimate of the meta-analyses (Figure 4: success after tympanic membrane reconstruction: comparison between patients with normal opposite ear status and patients with diseased opposite ear status). Significance levels are shaded in to help evaluate publication bias

## Slide 6
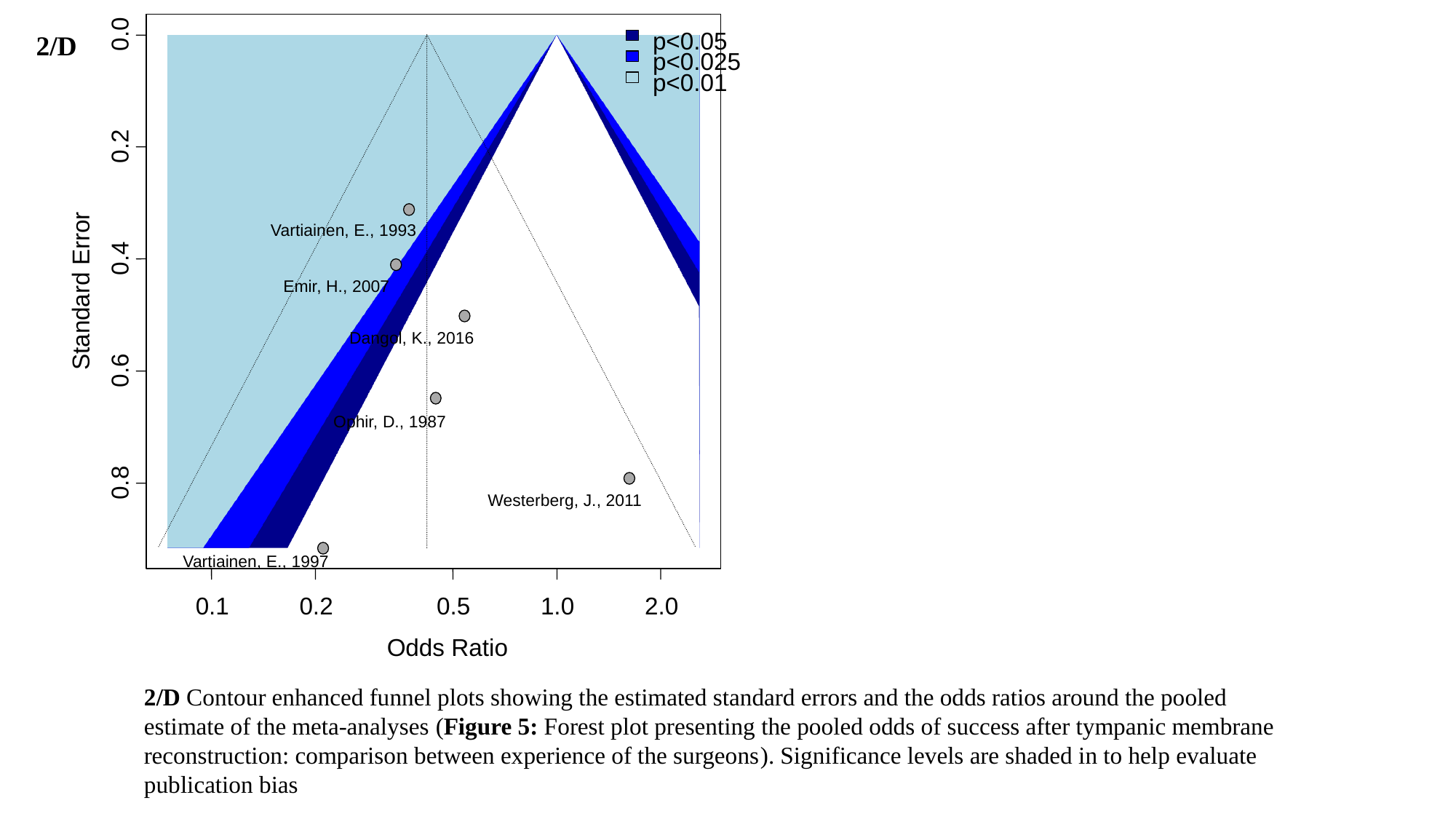

0.0
p<0.05
p<0.025
p<0.01
0.2
Vartiainen, E., 1993
0.4
Emir, H., 2007
Standard Error
Dangol, K., 2016
0.6
Ophir, D., 1987
0.8
Westerberg, J., 2011
Vartiainen, E., 1997
0.1
0.2
0.5
1.0
2.0
Odds Ratio
2/D
2/D Contour enhanced funnel plots showing the estimated standard errors and the odds ratios around the pooled estimate of the meta-analyses (Figure 5: Forest plot presenting the pooled odds of success after tympanic membrane reconstruction: comparison between experience of the surgeons). Significance levels are shaded in to help evaluate publication bias

## Slide 7
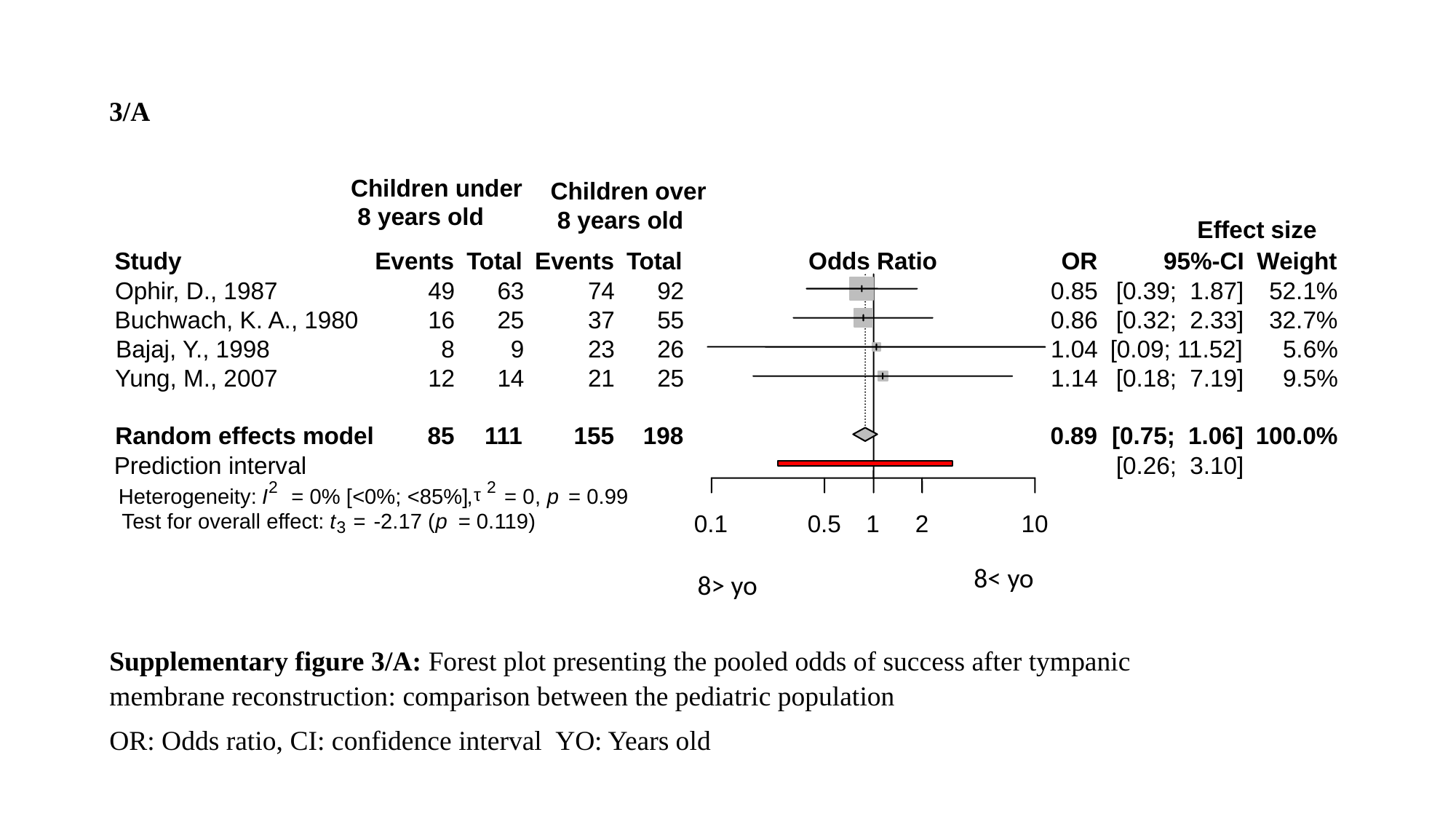

Children under
 8 years old
Children over
 8 years old
Study
Events
Total
Events
Total
Odds Ratio
OR
95%-CI
Weight
Ophir, D., 1987
49
63
74
92
0.85
[0.39; 1.87]
52.1%
Buchwach, K. A., 1980
16
25
37
55
0.86
[0.32; 2.33]
32.7%
Bajaj, Y., 1998
8
9
23
26
1.04
[0.09; 11.52]
5.6%
Yung, M., 2007
12
14
21
25
1.14
[0.18; 7.19]
9.5%
Random effects model
85
111
155
198
0.89
[0.75; 1.06]
100.0%
Prediction interval
[0.26; 3.10]
2
2
Heterogeneity:
I
 = 0% [<0%; <85%]
,
τ
 = 0
,
p
 = 0.99
Test for overall effect:
t
 =
-2.17
 (
p
 = 0.119
)
0.1
0.5
1
2
10
3
3/A
Effect size
8< yo
8> yo
Supplementary figure 3/A: Forest plot presenting the pooled odds of success after tympanic membrane reconstruction: comparison between the pediatric population
OR: Odds ratio, CI: confidence interval YO: Years old

## Slide 8
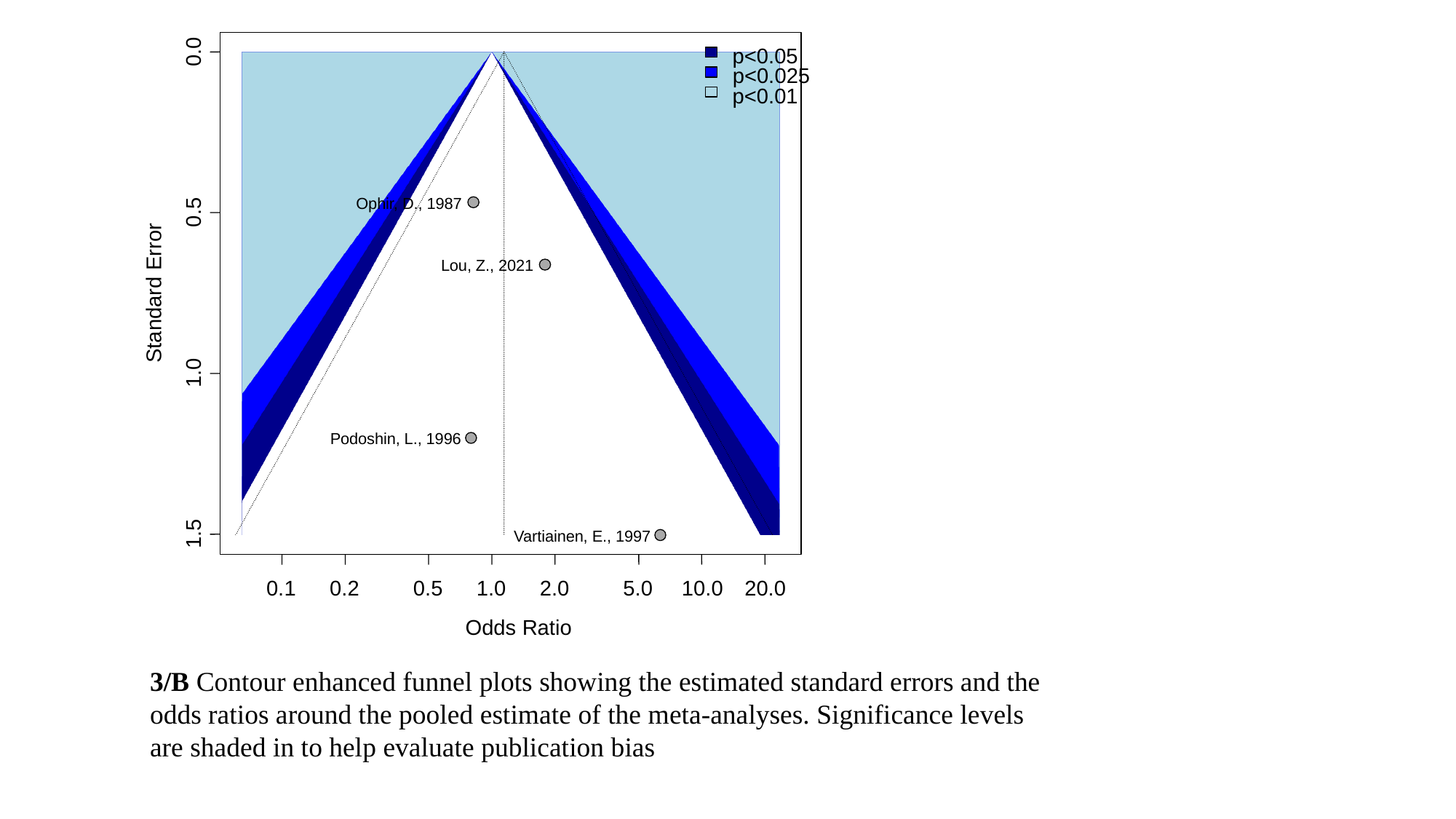

0.0
p<0.05
p<0.025
p<0.01
Ophir, D., 1987
0.5
Lou, Z., 2021
Standard Error
1.0
Podoshin, L., 1996
1.5
Vartiainen, E., 1997
0.1
0.2
0.5
1.0
2.0
5.0
10.0
20.0
Odds Ratio
3/B Contour enhanced funnel plots showing the estimated standard errors and the odds ratios around the pooled estimate of the meta-analyses. Significance levels are shaded in to help evaluate publication bias

## Slide 9
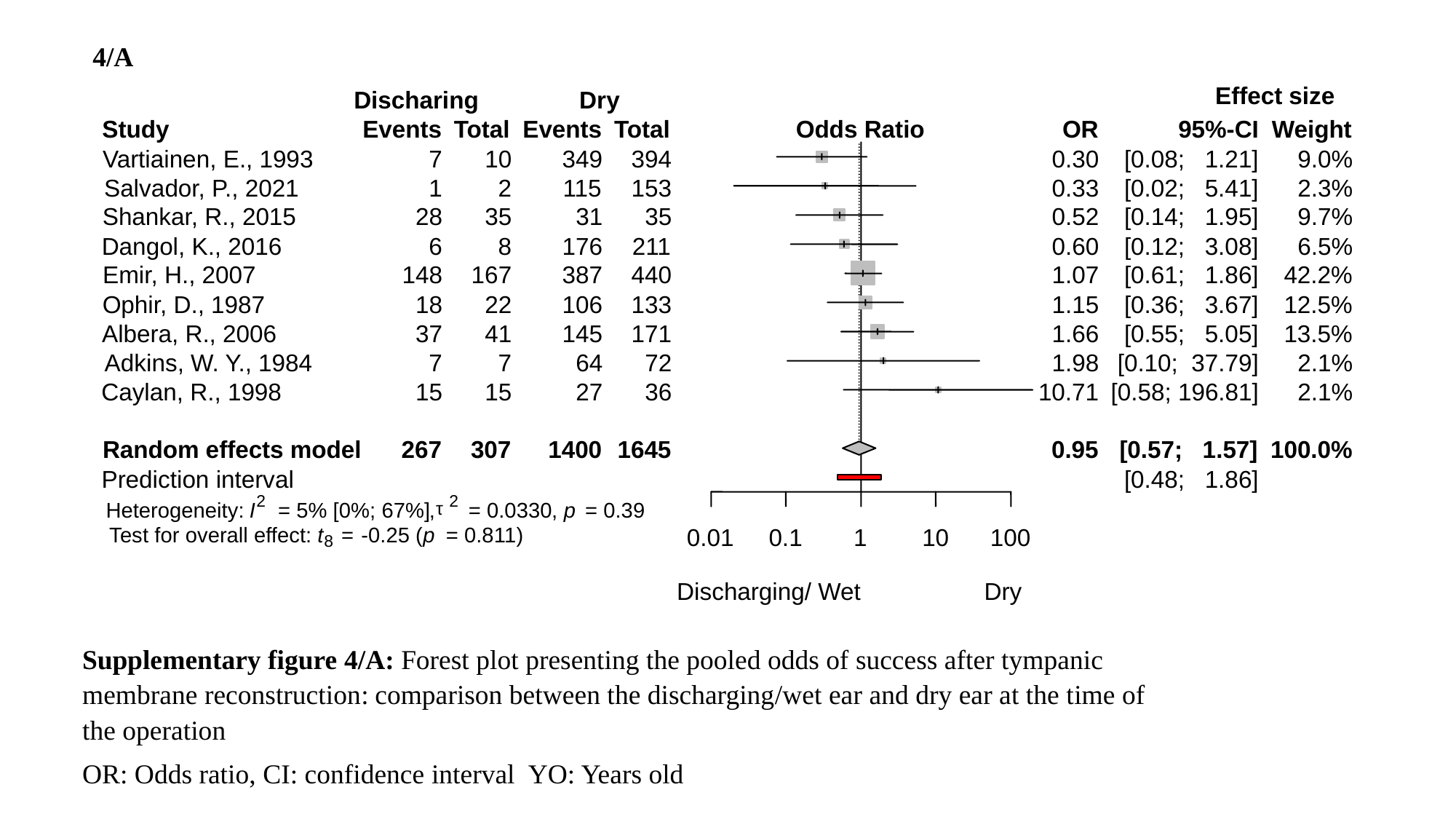

Discharing
Dry
Study
Events
Total
Events
Total
Odds Ratio
OR
95%-CI
Weight
Vartiainen, E., 1993
7
10
349
394
0.30
[0.08; 1.21]
9.0%
Salvador, P., 2021
1
2
115
153
0.33
[0.02; 5.41]
2.3%
Shankar, R., 2015
28
35
31
35
0.52
[0.14; 1.95]
9.7%
Dangol, K., 2016
6
8
176
211
0.60
[0.12; 3.08]
6.5%
Emir, H., 2007
148
167
387
440
1.07
[0.61; 1.86]
42.2%
Ophir, D., 1987
18
22
106
133
1.15
[0.36; 3.67]
12.5%
Albera, R., 2006
37
41
145
171
1.66
[0.55; 5.05]
13.5%
Adkins, W. Y., 1984
7
7
64
72
1.98
[0.10; 37.79]
2.1%
Caylan, R., 1998
15
15
27
36
10.71
[0.58; 196.81]
2.1%
Random effects model
267
307
1400
1645
0.95
[0.57; 1.57]
100.0%
Prediction interval
[0.48; 1.86]
2
2
Heterogeneity:
I
 = 5% [0%; 67%]
,
τ
 = 0.0330
,
p
 = 0.39
Test for overall effect:
t
 =
-0.25
 (
p
 = 0.811
)
0.01
0.1
1
10
100
8
4/A
Effect size
Discharging/ Wet
Dry
Supplementary figure 4/A: Forest plot presenting the pooled odds of success after tympanic membrane reconstruction: comparison between the discharging/wet ear and dry ear at the time of the operation
OR: Odds ratio, CI: confidence interval YO: Years old

## Slide 10
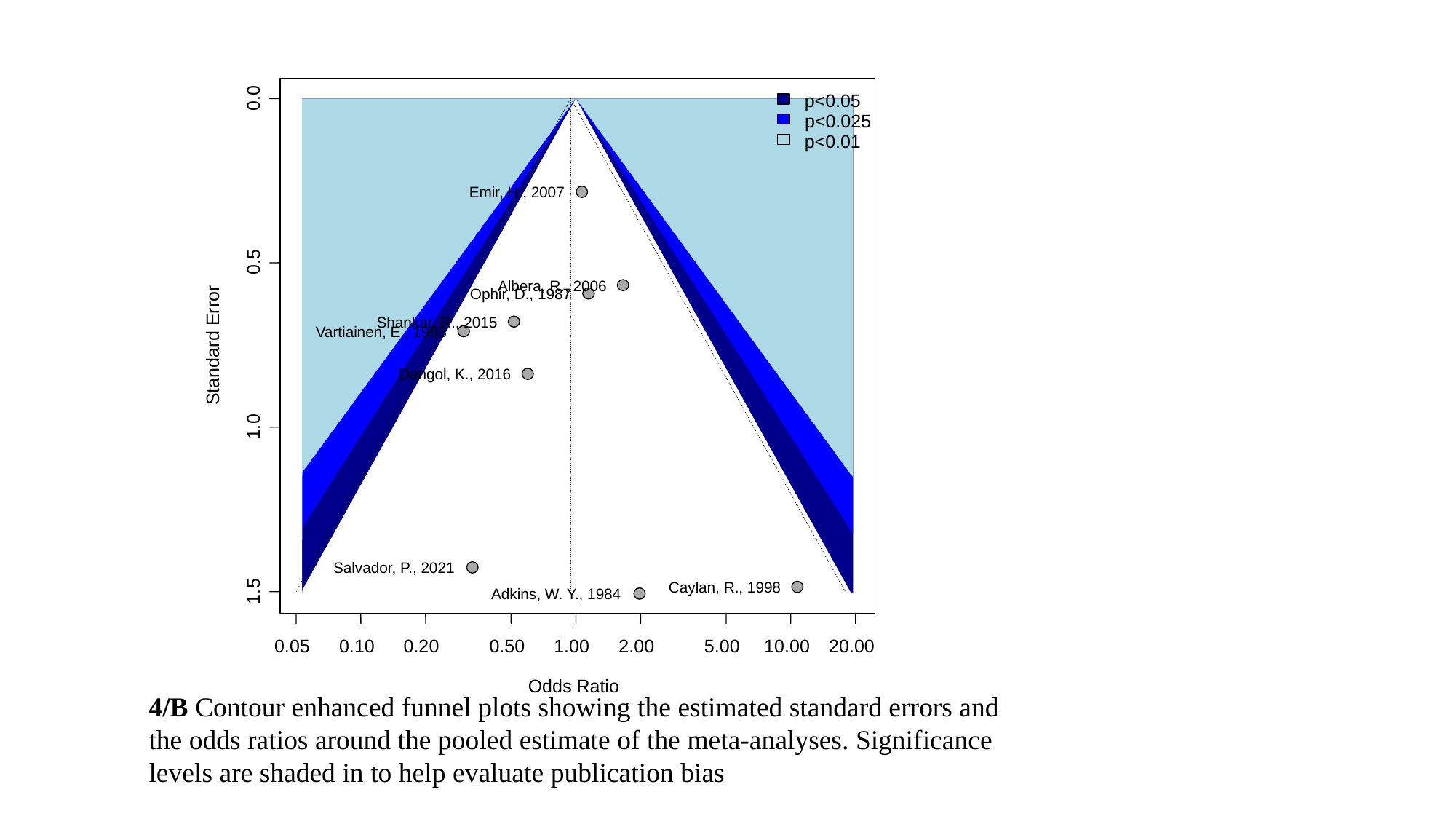

0.0
p<0.05
p<0.025
p<0.01
Emir, H., 2007
0.5
Albera, R., 2006
Ophir, D., 1987
Shankar, R., 2015
Vartiainen, E., 1993
Standard Error
Dangol, K., 2016
1.0
Salvador, P., 2021
Caylan, R., 1998
1.5
Adkins, W. Y., 1984
0.05
0.10
0.20
0.50
1.00
2.00
5.00
10.00
20.00
Odds Ratio
4/B Contour enhanced funnel plots showing the estimated standard errors and the odds ratios around the pooled estimate of the meta-analyses. Significance levels are shaded in to help evaluate publication bias

## Slide 11
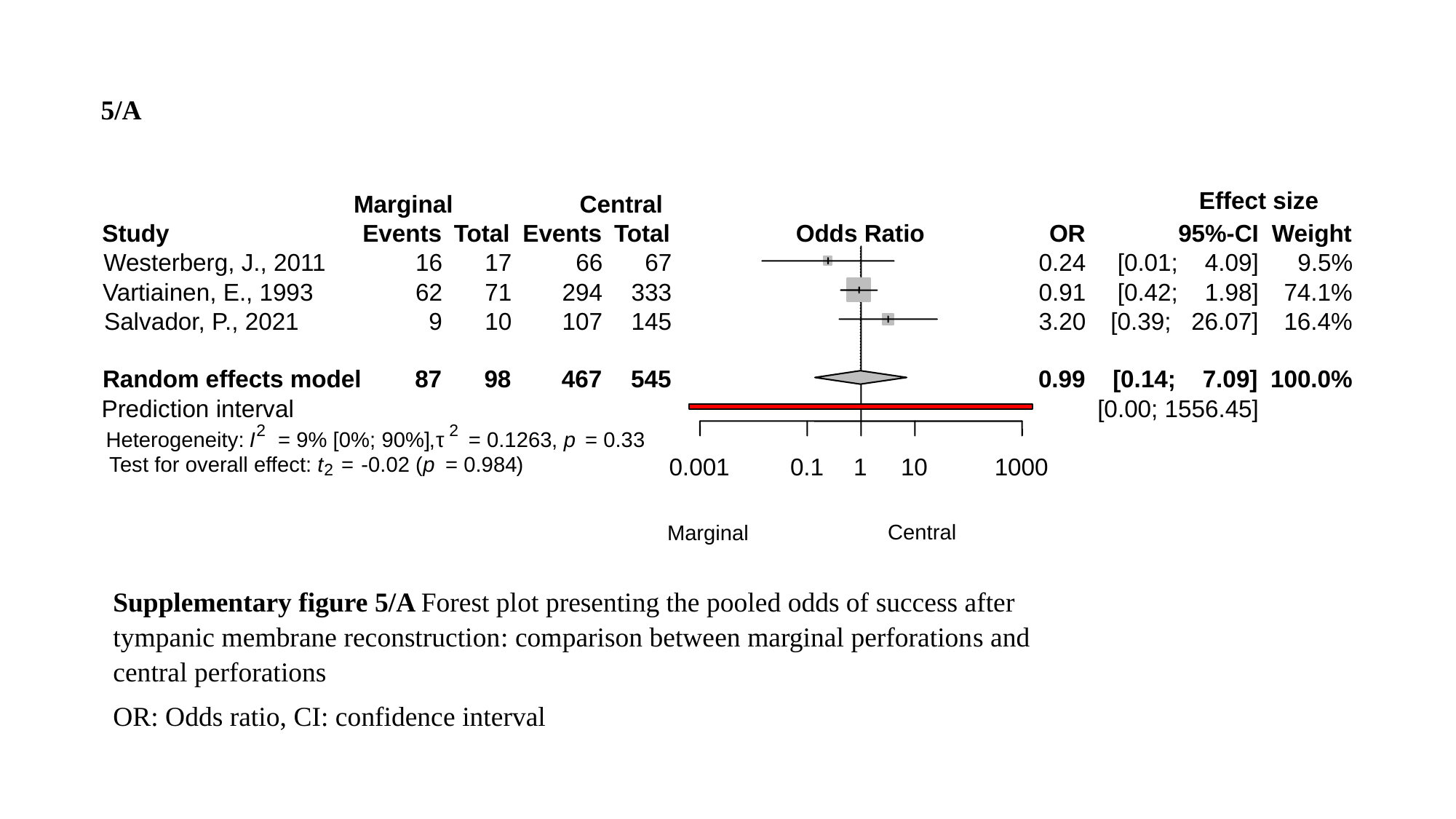

Marginal
Central
Study
Events
Total
Events
Total
Odds Ratio
OR
95%-CI
Weight
Westerberg, J., 2011
16
17
66
67
0.24
[0.01; 4.09]
9.5%
Vartiainen, E., 1993
62
71
294
333
0.91
[0.42; 1.98]
74.1%
Salvador, P., 2021
9
10
107
145
3.20
[0.39; 26.07]
16.4%
Random effects model
87
98
467
545
0.99
[0.14; 7.09]
100.0%
Prediction interval
[0.00; 1556.45]
2
2
Heterogeneity:
I
 = 9% [0%; 90%]
,
τ
 = 0.1263
,
p
 = 0.33
Test for overall effect:
t
 =
-0.02
 (
p
 = 0.984
)
0.001
0.1
1
10
1000
2
5/A
Effect size
Central
Marginal
Supplementary figure 5/A Forest plot presenting the pooled odds of success after tympanic membrane reconstruction: comparison between marginal perforations and central perforations
OR: Odds ratio, CI: confidence interval

## Slide 12
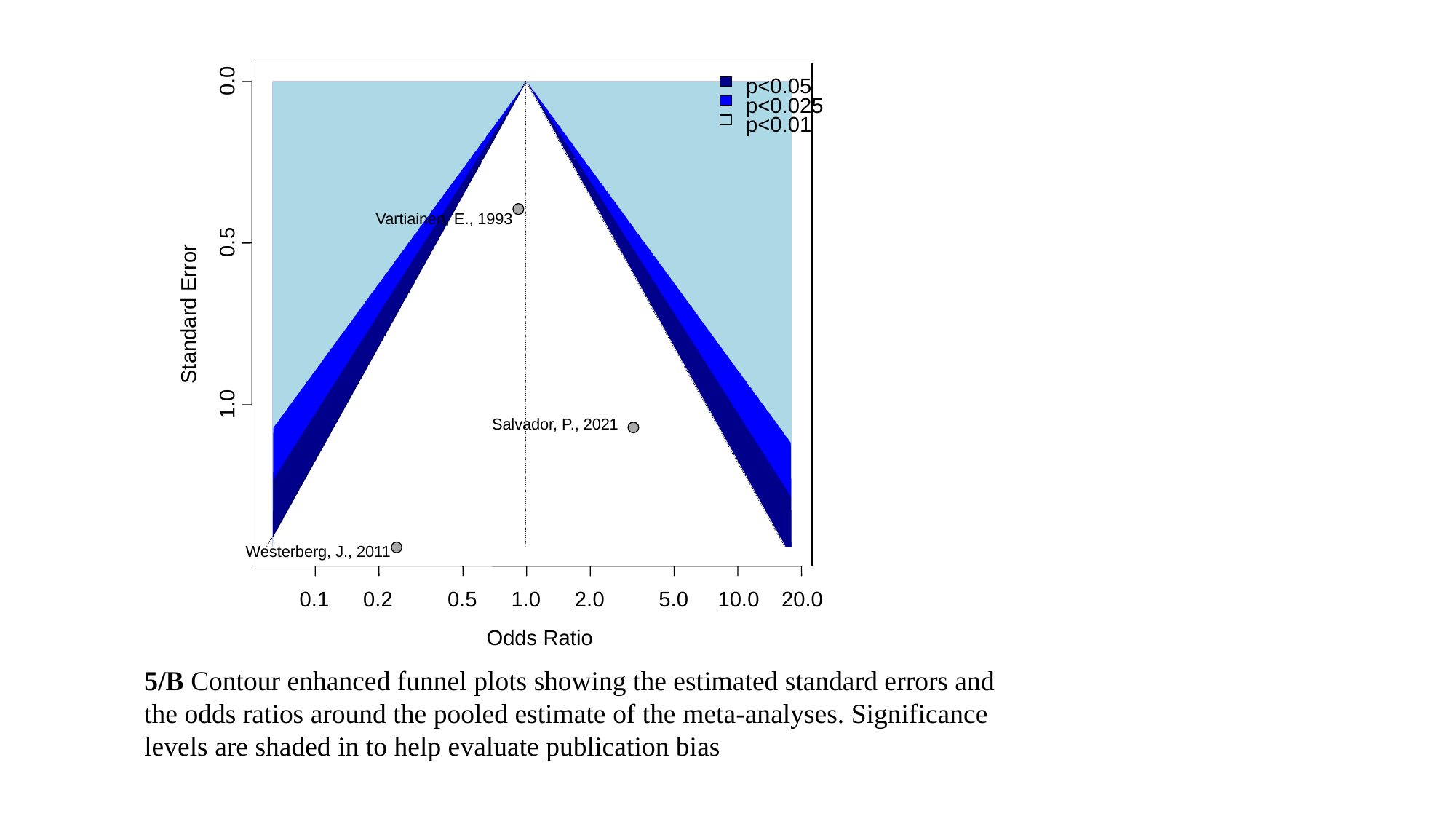

0.0
p<0.05
p<0.025
p<0.01
Vartiainen, E., 1993
0.5
Standard Error
1.0
Salvador, P., 2021
Westerberg, J., 2011
0.1
0.2
0.5
1.0
2.0
5.0
10.0
20.0
Odds Ratio
5/B Contour enhanced funnel plots showing the estimated standard errors and the odds ratios around the pooled estimate of the meta-analyses. Significance levels are shaded in to help evaluate publication bias

## Slide 13
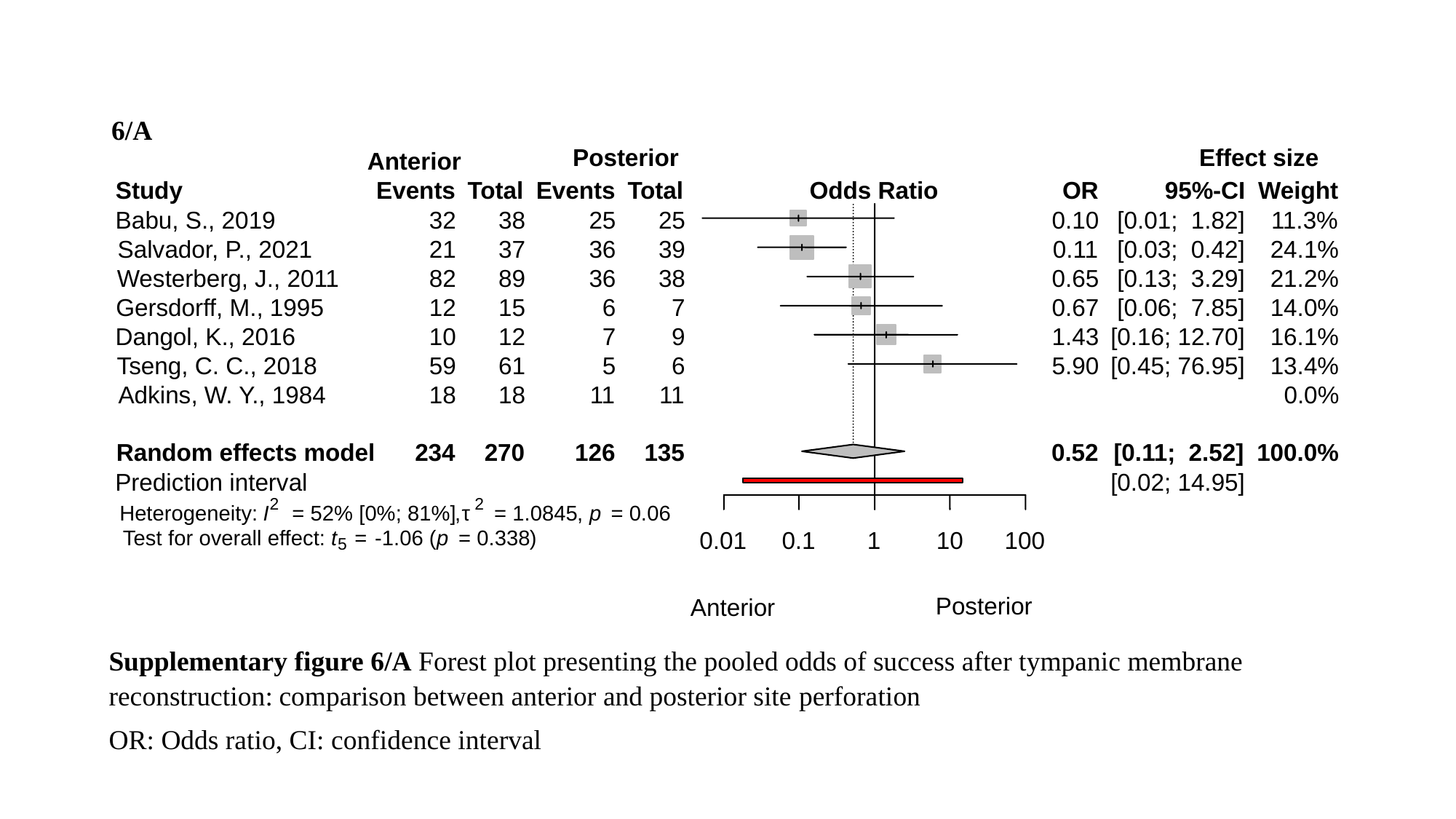

Posterior
Anterior
Study
Events
Total
Events
Total
Odds Ratio
OR
95%-CI
Weight
Babu, S., 2019
32
38
25
25
0.10
[0.01; 1.82]
11.3%
Salvador, P., 2021
21
37
36
39
0.11
[0.03; 0.42]
24.1%
Westerberg, J., 2011
82
89
36
38
0.65
[0.13; 3.29]
21.2%
Gersdorff, M., 1995
12
15
6
7
0.67
[0.06; 7.85]
14.0%
Dangol, K., 2016
10
12
7
9
1.43
[0.16; 12.70]
16.1%
Tseng, C. C., 2018
59
61
5
6
5.90
[0.45; 76.95]
13.4%
Adkins, W. Y., 1984
18
18
11
11
0.0%
Random effects model
234
270
126
135
0.52
[0.11; 2.52]
100.0%
Prediction interval
[0.02; 14.95]
2
2
Heterogeneity:
I
 = 52% [0%; 81%]
,
τ
 = 1.0845
,
p
 = 0.06
Test for overall effect:
t
 =
-1.06
 (
p
 = 0.338
)
0.01
0.1
1
10
100
5
6/A
Effect size
Posterior
Anterior
Supplementary figure 6/A Forest plot presenting the pooled odds of success after tympanic membrane reconstruction: comparison between anterior and posterior site perforation
OR: Odds ratio, CI: confidence interval

## Slide 14
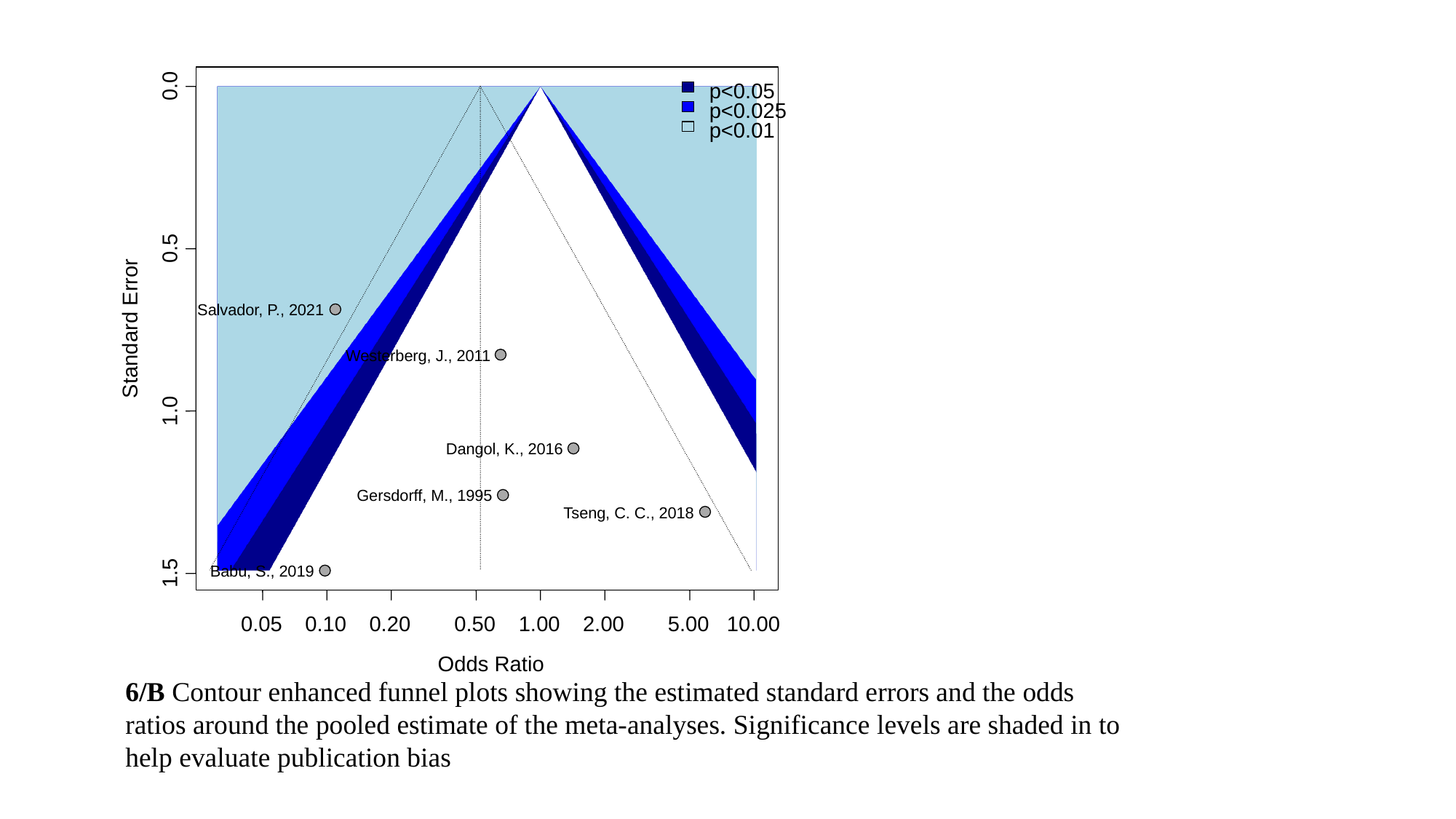

0.0
p<0.05
p<0.025
p<0.01
0.5
Salvador, P., 2021
Standard Error
Westerberg, J., 2011
1.0
Dangol, K., 2016
Gersdorff, M., 1995
Tseng, C. C., 2018
1.5
Babu, S., 2019
0.05
0.10
0.20
0.50
1.00
2.00
5.00
10.00
Odds Ratio
6/B Contour enhanced funnel plots showing the estimated standard errors and the odds ratios around the pooled estimate of the meta-analyses. Significance levels are shaded in to help evaluate publication bias

## Slide 15
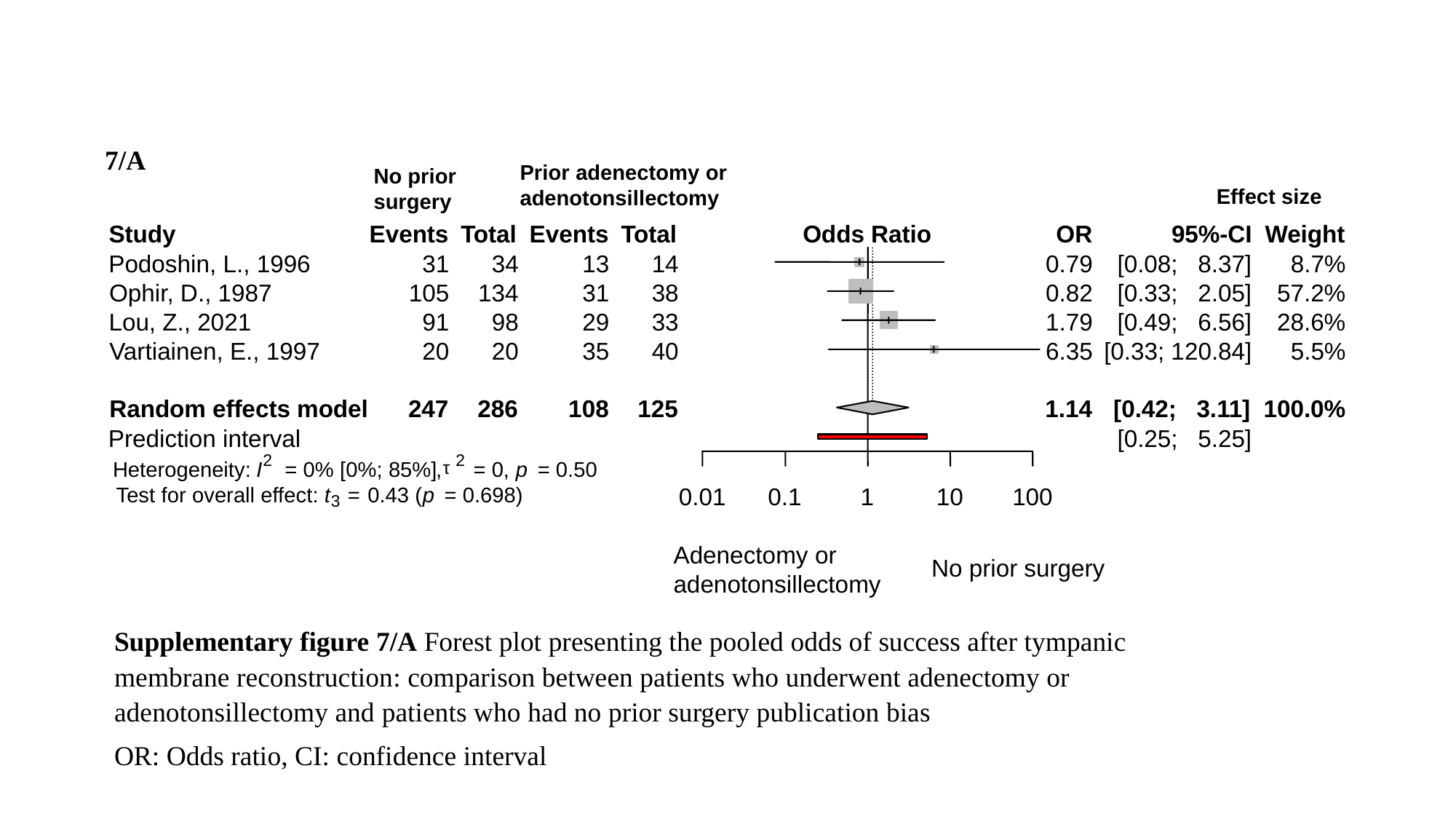

Prior adenectomy or adenotonsillectomy
No prior surgery
Study
Events
Total
Events
Total
Odds Ratio
OR
95%-CI
Weight
Podoshin, L., 1996
31
34
13
14
0.79
[0.08; 8.37]
8.7%
Ophir, D., 1987
105
134
31
38
0.82
[0.33; 2.05]
57.2%
Lou, Z., 2021
91
98
29
33
1.79
[0.49; 6.56]
28.6%
Vartiainen, E., 1997
20
20
35
40
6.35
[0.33; 120.84]
5.5%
Random effects model
247
286
108
125
1.14
[0.42; 3.11]
100.0%
Prediction interval
[0.25; 5.25]
2
2
Heterogeneity:
I
 = 0% [0%; 85%]
,
τ
 = 0
,
p
 = 0.50
Test for overall effect:
t
 =
0.43
 (
p
 = 0.698
)
0.01
0.1
1
10
100
3
7/A
Effect size
Adenectomy or adenotonsillectomy
No prior surgery
Supplementary figure 7/A Forest plot presenting the pooled odds of success after tympanic membrane reconstruction: comparison between patients who underwent adenectomy or adenotonsillectomy and patients who had no prior surgery publication bias
OR: Odds ratio, CI: confidence interval

## Slide 16
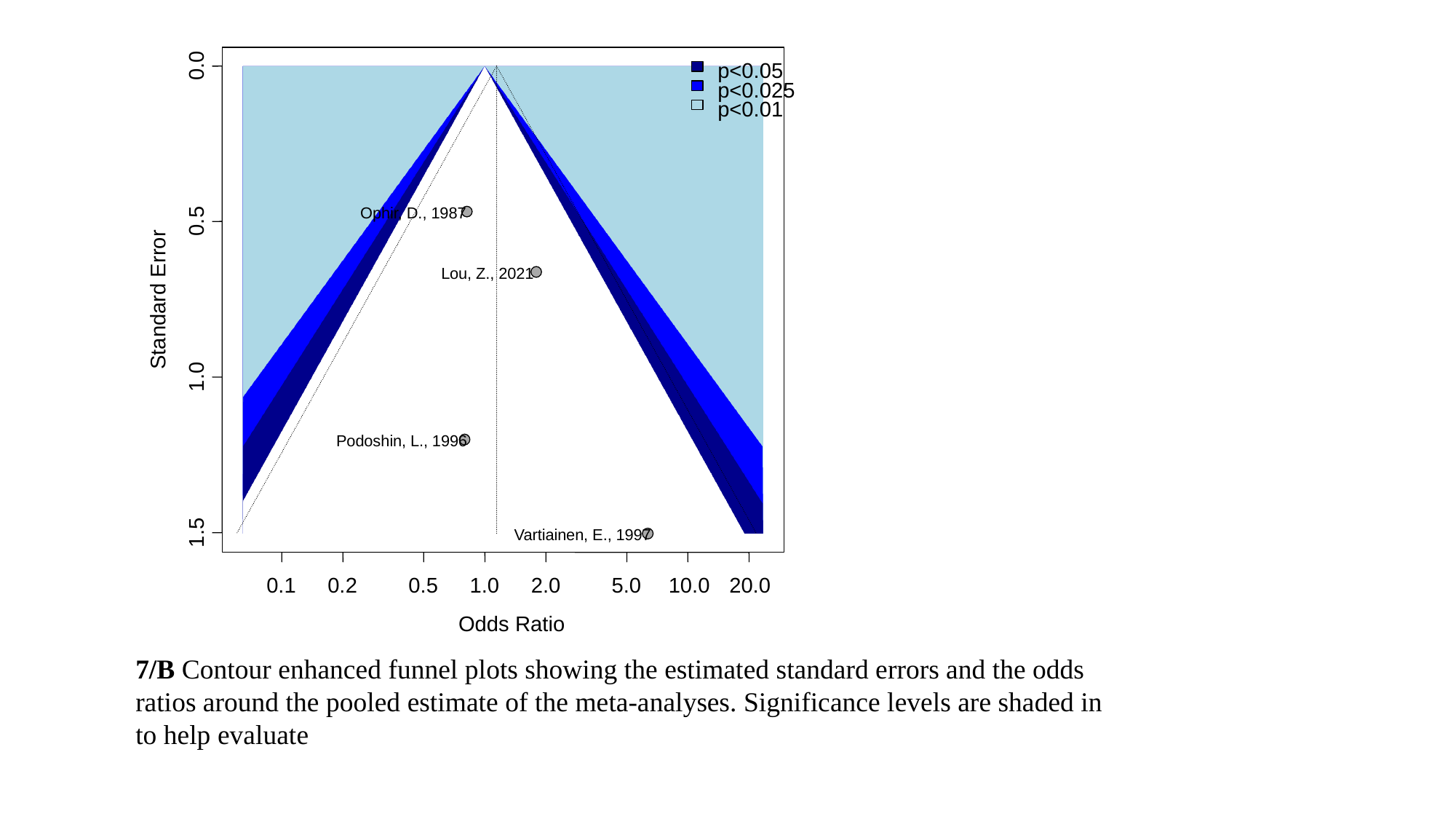

0.0
p<0.05
p<0.025
p<0.01
Ophir, D., 1987
0.5
Lou, Z., 2021
Standard Error
1.0
Podoshin, L., 1996
1.5
Vartiainen, E., 1997
0.1
0.2
0.5
1.0
2.0
5.0
10.0
20.0
Odds Ratio
7/B Contour enhanced funnel plots showing the estimated standard errors and the odds ratios around the pooled estimate of the meta-analyses. Significance levels are shaded in to help evaluate

## Slide 17
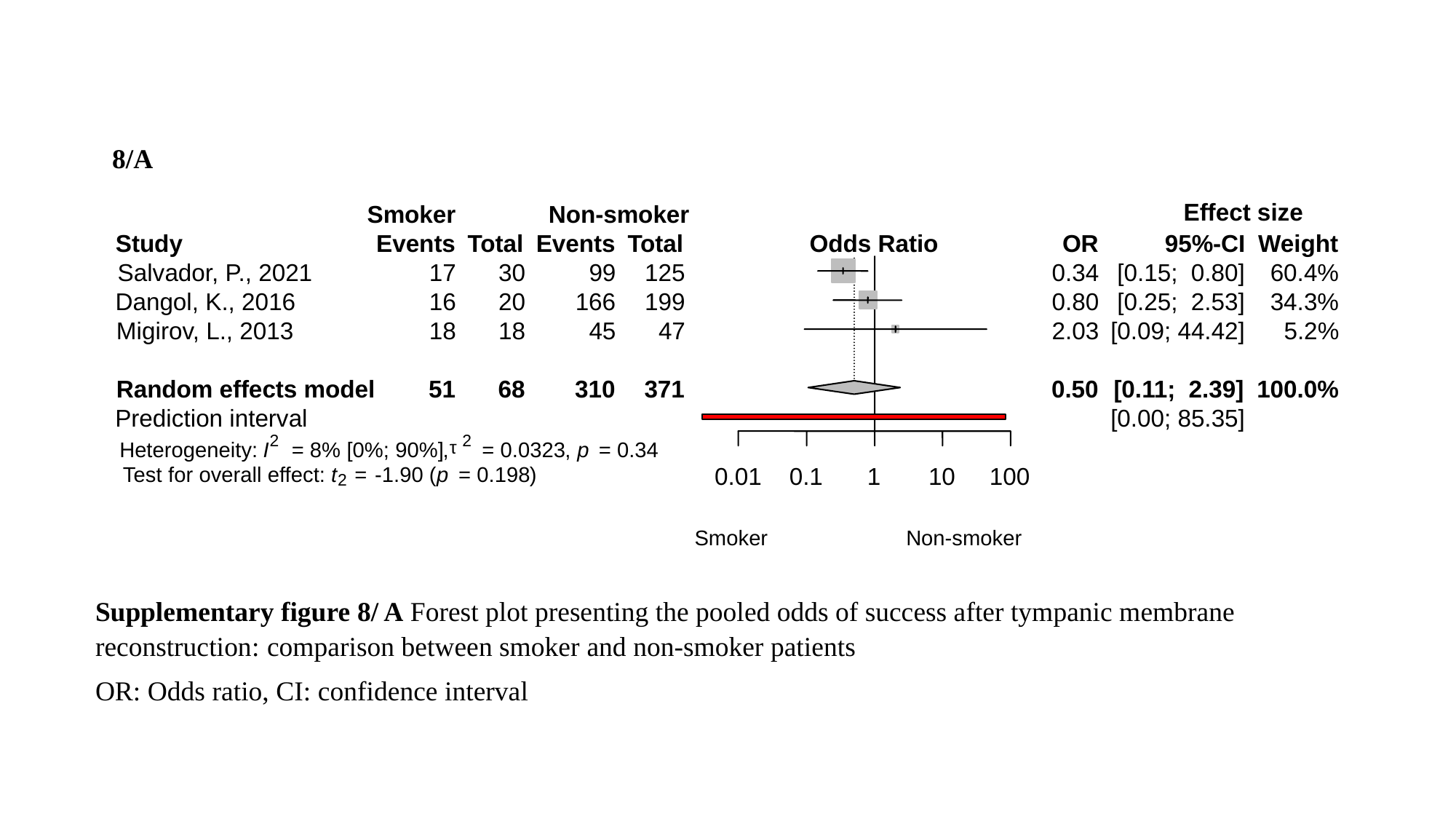

Smoker
Non-smoker
Study
Events
Total
Events
Total
Odds Ratio
OR
95%-CI
Weight
Salvador, P., 2021
17
30
99
125
0.34
[0.15; 0.80]
60.4%
Dangol, K., 2016
16
20
166
199
0.80
[0.25; 2.53]
34.3%
Migirov, L., 2013
18
18
45
47
2.03
[0.09; 44.42]
5.2%
Random effects model
51
68
310
371
0.50
[0.11; 2.39]
100.0%
Prediction interval
[0.00; 85.35]
2
2
Heterogeneity:
I
 = 8% [0%; 90%]
,
τ
 = 0.0323
,
p
 = 0.34
Test for overall effect:
t
 =
-1.90
 (
p
 = 0.198
)
0.01
0.1
1
10
100
2
8/A
Effect size
Smoker
Non-smoker
Supplementary figure 8/ A Forest plot presenting the pooled odds of success after tympanic membrane reconstruction: comparison between smoker and non-smoker patients
OR: Odds ratio, CI: confidence interval

## Slide 18
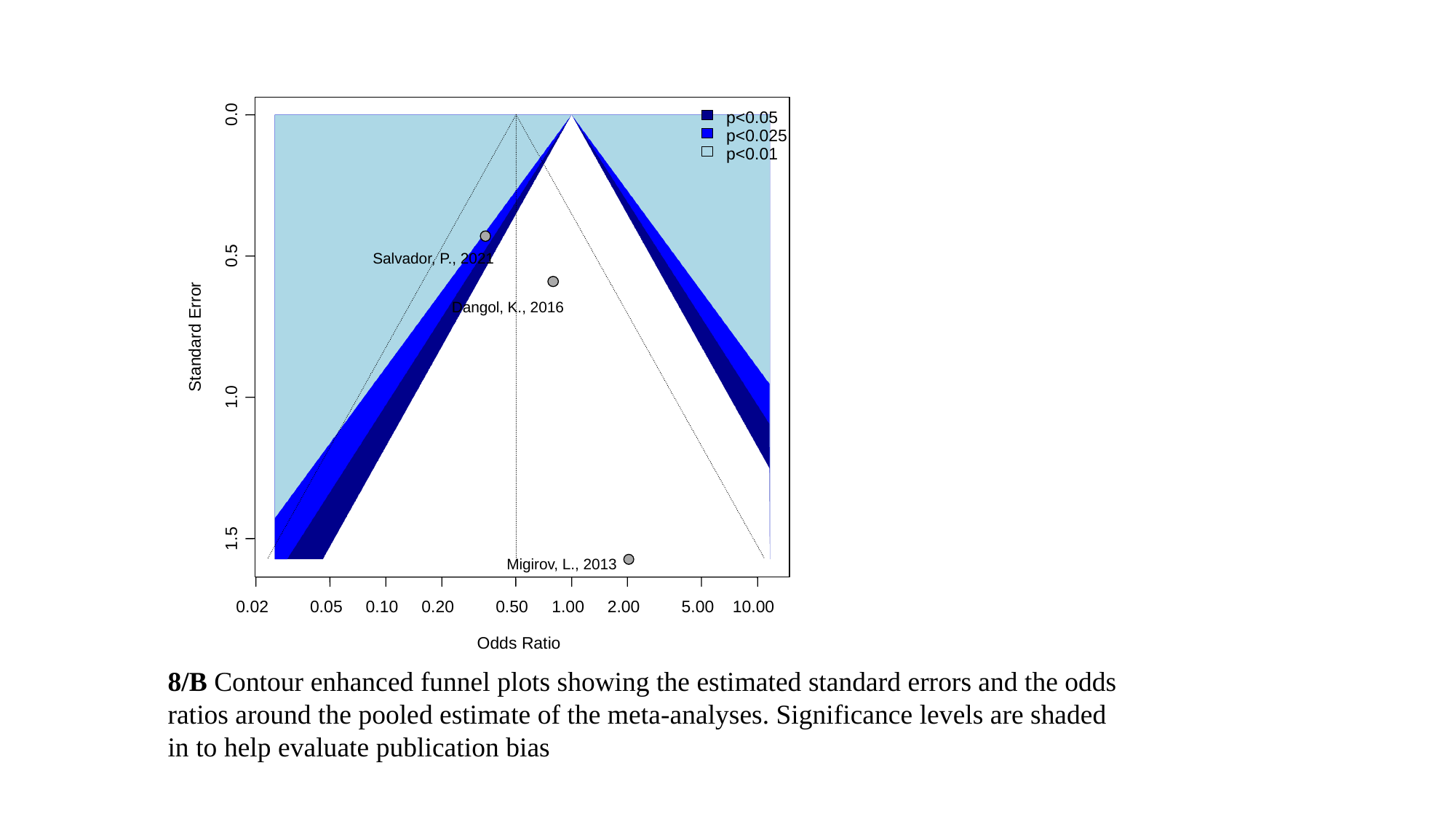

0.0
p<0.05
p<0.025
p<0.01
0.5
Salvador, P., 2021
Dangol, K., 2016
Standard Error
1.0
1.5
Migirov, L., 2013
0.02
0.05
0.10
0.20
0.50
1.00
2.00
5.00
10.00
Odds Ratio
8/B Contour enhanced funnel plots showing the estimated standard errors and the odds ratios around the pooled estimate of the meta-analyses. Significance levels are shaded in to help evaluate publication bias
